# Primary support optimization of large-span and shallow buried hard rock tunnels based on the active support concept

**DOI:** 10.1038/s41598-022-11399-y

**Published:** 2022-05-13

**Authors:** Quanwei Liu, Yongshun Li, Weiteng Li, Jizeng Zhao, Zhe Qin, Xuxu Yang, Qiang Feng, Bei Jiang, Ke Wang, Yang Li

**Affiliations:** 1 Qingdao Metro Group Corporation, Qingdao, 266000 China; 2 Shandong Key Laboratory of Civil Engineering Disaster Prevention and Reduction, Shandong University of Science and Technology, Qingdao, 266590 Shandong China; 3 State Key Laboratory for Geo-Mechanics and Deep Underground Engineering, China University of Mining and Technology-Beijing, Beijing, 100083 China

**Keywords:** Engineering, Civil engineering

## Abstract

Considering the large-span underground excavation subway station of Qingdao Metro Line 6 of China for analysis, it is necessary to optimize the traditional support system by investigating relevant codes and other tunnel projects. Based on the active support concept, a high prestressed rock bolt support system is proposed, and the optimization direction is defined to apply a high prestress force to rock bolts, increasing the appropriate spacing between supporting arches and strengthening support at key parts such as the large arch foot area, sidewalls and junctions. Numerical calculations and field monitoring are performed to analyze and evaluate the new support system. Numerical simulation results show that the new support system can effectively improve the stress state of the surrounding rock; the tensile stress area markedly decreases in size or disappears; and the plastic area also decreases in size. Field monitoring results show that the settlement of the arch crown is concentrated at 2–5 mm and the deformation rates are less than 0.5 mm/day. The supporting arches, shotcrete and rock bolts are all less than the yield strength and a high safety reserve. These results verify the safety and rationality of the proposed support system, which can be used as a reference for similar projects.

## Introduction

With the sustained and healthy development of China's economy and the continuous improvement of China's comprehensive strength, the real demand and engineering technology of transportation construction have made major breakthroughs, and China's tunnel construction has achieved unprecedented development. China already has the largest construction industry, the most tunnels, the most complex construction conditions, and the fastest construction speeds in the world. Many large-span tunnels have also been built that have generally used composite linings, and their initial support generally uses the combined support of bolts, shotcrete and steel frames.

With the increasing number of engineering constructions and wide variety of working conditions, some problems in the initial support design and construction of tunnels have gradually identified. The low effectiveness of bolt support systems is most prominent^1–3^. In railway tunnel engineering, full-length bonded non-prestressed rock bolts are generally used, but the supporting effect of the rock bolts is unclear. Under what working conditions rock bolts can work or not remains vague, particularly in the arch joint supporting system; their supporting effect remains poorly understood. In addition, tunnel bolts generally use hollow grouting bolts. Arch grouting is difficult, particularly when applied prestressed. The quality of rock bolts and construction quality are not verifiable, and the expected active support effect cannot be achieved.

Many scholars have investigated the role of rock bolts. Xia^4^ improved the tunnel anchorage coordination theory and proposed an improved synergy mechanism. Zhang^5^ analyzed the mechanical characteristics and load effect of tunnel’s surrounding rock; established the structural mechanical model of deep and shallow surrounding rock; and proposed the perspective that tunnel bolt supports have the basic function of mobilizing and assisting the bearing of surrounding rock. Pinazzi^6^ proposed the failure criterion of bolts under a combined load, which provides guidance for the design of bolt bearing capacity. Sun^7^ proposed a method for determining tunnel anchorage parameters based on the synergy of anchorage systems, which provides a theoretical basis for the quantitative design of anchorage systems in tunnel and underground engineering. Xu^8^ compared the anchoring effects of prestressed bolts and fully bonded bolts on fractured rock masses, and determined the influence of the two types of bolts on the mechanical behavior of fractured rock masses. Skrzypkowski^9^ studied the stress–strain characteristics of various bolt support forms in order to correctly select the bolt support form according to the specific geological conditions in order to give full play to the bolt effect. Yan^10^ deduced the analytical solution of the characteristic curve of the surrounding rock of the composite rock mass of the circular tunnel with bolt reinforcement. By comparing the characteristic curve of the surrounding rock with or without bolt support, the bolt achieved good control of the deformation of the surrounding rock. Liu^11^ developed an anchoring cooperative component group to optimize the bolt anchoring structure and improve the bolt anchoring capacity to control the surrounding rock deformation. Mohammad Mohammadi^12^ established the mathematical theory of the bolt support mechanism using the bolt support coefficient, which provided a new theoretical basis for explaining the bolt support effect, and defined the anchorage reinforcement capacity of a given rock mass using the discontinuity parameters in the Q-system.

Underground excavated subway stations are essentially tunnels that are generally buried shallow, have large spans, and require higher safety restrictions in urban construction. Qingdao is composed of a typical hard rock stratum, and underground excavation methods are primarily used in subway station construction. The underground excavation station has a large span, is composed of hard rock, is buried shallow, and has distinctive characteristics at home and abroad. Based on the engineering background of the large-span hard rock shallow buried tunnel of the underground excavation station of Qingdao Metro, the current use of conventional hollow grouting bolts is systematically organized, and based on the analysis of the prestressed rock bolt support mechanism, the stress–strain characteristics of rock bolt support are analyzed, a prestressed rock bolt active support system is proposed in this study. Therefore, we aim to investigate relevant codes and other tunnel projects to optimize the scheme design, perform field practice and monitoring, analyze and evaluate the new support system.

## Project overview

Qingdao is an economic center, a modern service center, and a cultural center of the eastern coastal region of China. To adapt to changes in the urban scheme, Qingdao planned 29 subway lines with a total length of approximately 1232 km. The total length of Metro Line 6 is 57.56 km, and 38 stations are set along the entire line, including 6 underground excavation stations.

**Figure 1:**
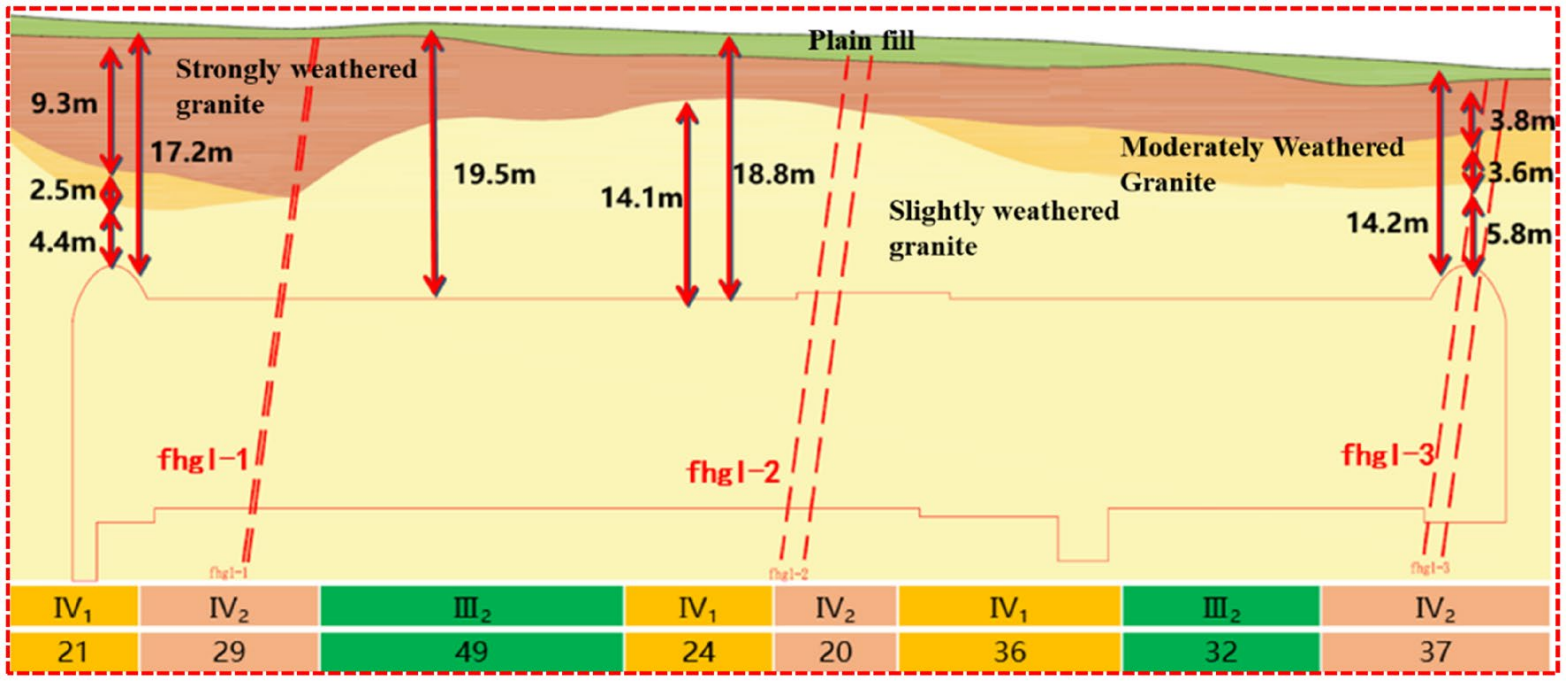
Geological date of underground excavation subway station.

Considering an underground excavation subway stations as an example (see Fig. 1 for geological data), the geology of the site is composed of a plain soil layer, a strongly weathered granite layer, sandy cataclastic rock layer, moderately weathered granite layer, marginally weathered granite layer, and marginally weathered granite porphyry layer from the ground surface. There are three fracture zones within the station, numbered fhg I-1, fhg I-2 and fhg I-3 respectively. The strike of the three fracture zones is approximately NE and the width is about 3–5 m. Among them, the surrounding rock of fhg I-2 and fhg I-3 is relatively broken. The surrounding rock grade is grade III2 ~ IV2. The primary structure of the station is located in a marginally weathered rock stratum, the surrounding rock is relatively broken, and the geotechnical parameters are shown in Table 1. The groundwater is primarily bedrock fissure water, and the water volume is generally poor.

**Table 1:** Rock parameter table of subway station.

Stratum	Elastic modulus/10^3^ MPa	Poisson's ratio	Cohesion force/MPa	Internal friction angle/(°)	Natural density g/cm^3^
Plain soil	–	–	–	18	1.80
Strongly weathered granite	–	0.24	–	45	2.35
Sandy fragmented rock	–	0.25	–	40	2.25
Moderately weathered granite	6.0	0.22	–	55	2.45
Marginally weathered granite	31.9	0.21	7.74	65	2.56
Lightly weathered granite porphyry	37.7	0.20	8.29	65	2.60

## Traditional excavation support scheme

### Traditional excavation scheme

The commonly used construction methods of underground excavation subway stations in strata include the double-side-drift method and arch cover method.

**Figure 2:**
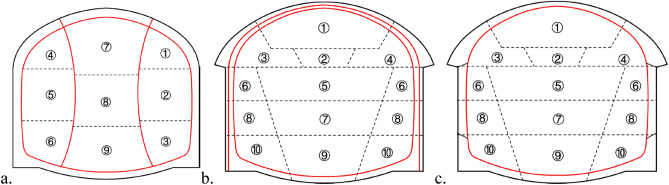
Comparison of the double-side-drift method and arch cover method. (**a**) The double-side-drift method; (**b**) the primary support arch cover method (PCM); (**c**) the secondary lining arch cover method (SCM).

The double-side-drift method is primarily applicable to subway projects with poor strata and large sections. The excavation section is divided into many blocks with a large disturbance and long closing time of the entire section of the initial support. The arch cover method is primarily applicable to subway projects with large cross-sections, which form the upper arch cover through the supporting structure and then excavate the lower part of the station. The arch cover method is divided into the primary support arch cover method (PCM) and the secondary lining arch cover method (SCM). The SCM forms the arch cover structure system through the permanent secondary lining structure, while the PCM through the primary support. The comparison of above construction methods is shown in Fig. 2, where the digits represent the excavation steps.

The quality of the surrounding rock in the Qingdao area is good. However, the primary structure of the tunnel is a long-span single arch structure; the upper soft soil layer and broken rock layer are more sensitive to uneven settlement; and the conditions at the interface of auxiliary structures such as air ducts and entrances and exits are complex. According to the above comparison and in combination with the geological exploration data, the station uses SCM for construction.

### Traditional support scheme

**Figure 3:**
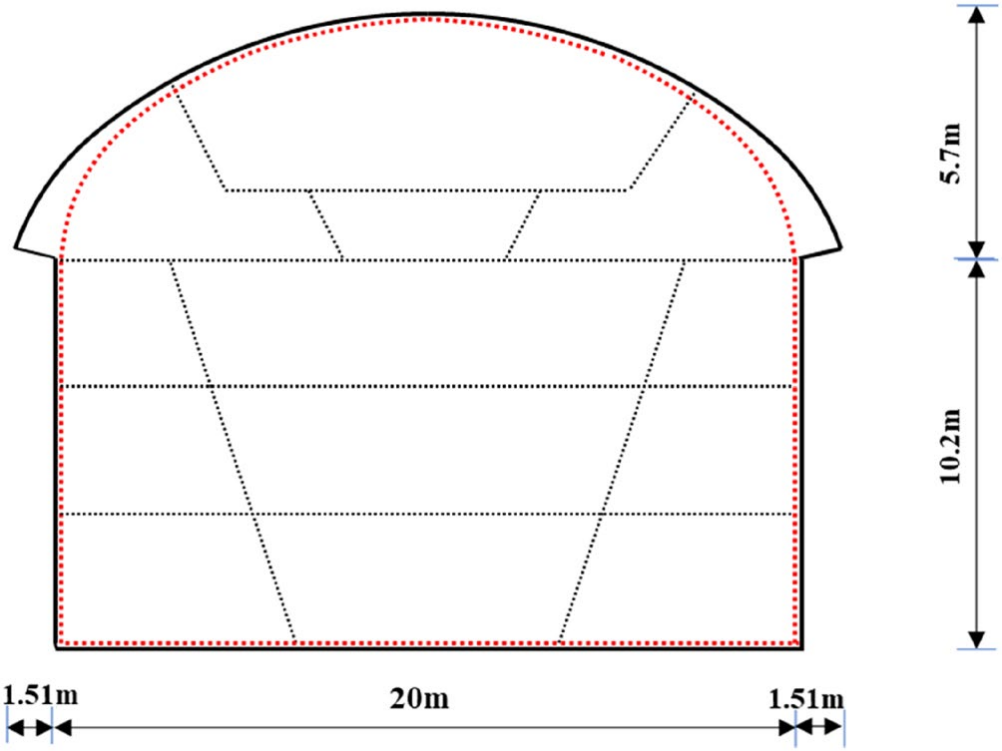
A section of the primary structure of the station.

The primary structure of the station is primarily located in marginally weathered granite and uses a single arch straight wall composite lining structure. The burial depth of the arch crown is approximately 14.8–19.9 m, and the overburden thickness is 13–27 m. The excavation span of the arch is approximately 23 m, the height is approximately 5.7 m, the rise span ratio is approximately 0.25, and the width of the large arch foot is 1.51 m. The excavation width of the lower section of the primary structure is 20 m, and the height is approximately 10.2 m. The section of the station is shown in Fig. 3, and the support parameters under the surrounding rock of different grades are shown in Table 2.

**Table 2:** Supporting parameters of primary structure.

Grade of surrounding rock	Shotcrete thickness at arch and grid spacing	Arch part φ25 hollow grouting rock bolt L = 4 m (ring × longitudinal)	Sidewall shotcrete thickness	Sidewall upper support φ25 hollow grouting rock bolt L = 4.5 m (ring × longitudinal)	Sidewall lower support φ25 hollow grouting bolt L = 3.5 m (ring × longitudinal)
Grade III2	350 mm, 0.75 m	1.5 m × 0.75 m	150 mm	1.0 m × 1.0 m	1.5 m × 1.5 m
Grade IV1	350 mm, 0.5 m	1.0 m × 1.0 m	150 mm	1.0 m × 1.0 m	1.2 m × 1.2 m
Grade IV2	350 mm, 0.5 m	1.2 m × 0.5 m	150 mm	1.0 m × 1.0 m	1.2 m × 1.0 m

### Evaluation of supporting scheme

Based on the analysis of the engineering conditions of the underground excavation station of Line 6, combined with the preliminary judgment of the engineering experience, the proposed scheme is conservative; however, potential problems of material waste are evident. Therefore, a systematic investigation and analysis of the proposed scheme was performed.

#### Investigation of relevant codes

The *Code for Design of Road Tunnel (in China)*^13^ provides a design method for system bolts, which is based on engineering analogy and supplemented by calculation and uses dynamic design. However, the maximum design span is a three-lane tunnel, which is smaller than the span of a subsurface excavation station and is therefore of little importance in this study. The *Guidelines for Design of Highway Tunnels (in China)*^14^ provides suggestions about the support parameters of a four-lane tunnel with a span similar to that of an underground excavation station. The spacing between arches is 0.75–1 m. The *Code for Design of Railway Tunnel (in China)*^15^ suggests that the design parameters should be determined according to the engineering analogy method, and no arches should be provided for level-III surrounding rock. The *Code for Design of Metros (in China)*^16^ does not provide restrictions on support parameters but stipulates that the design and construction parameters should be optimized through dynamic monitoring of surrounding rock and support. The *Technical Code For Engineering Ground Anchorages and Shotcrete Support (in China)*^17^ stipulates that the distance between bolts should not be greater than 1/2 of the length of the rock bolts. The *Norwegian Method of Tunneling* stipulates that no arches shall be provided for level-III surrounding rocks. Standardized survey statistics are shown in Tables 3 and 4. Two points of view are indicated based on these analyses: (1) the original design is based on the specification, which considers the high risk of subway stations, and the value is generally equal to or exceeds the recommended limit of the specification; and (2) the specification and the original design emphasize engineering analogy, but the quantitative analogy is in the specific design It is difficult to put it in place.

**Table 3:** Suggestions on initial support parameters given in the codes for surrounding rock of grade III.

Specification	Supporting arch		Rock bolt	
	Spray layer thickness(cm)	Spacing (m)	Spacing (m)	Length (m)
Code for design of road tunnel (three lanes)	12–20	1–1.2 (supporting arch is optional)	1.0–1.2	2.5–3.5
Guidelines for design of highway tunnel (four lanes)	20	1–1.2	1.0–1.2	3.0–5.0
Code for design of railway tunnel	10–12	Without arch	1.2–1.5	2.5–3.0
Code for design of metro	No restrictions and recommendations on supporting parameters are given. "Shotcrete, steel arches or rock bolts should be used as the primary supporting means."			
Technical code for engineering of ground anchorages and shotcrete support	15–20	/	The distance between rock bolts should not be greater than 1/2 of the rock bolt length	
Norwegian method of tunneling	5–9	Without supporting arch	2.2–2.5	5

**Table 4:** Suggestions on initial support parameters given in the codes for level-IV surrounding rock.

Specification	Supporting arch		Rock bolt	
	Spray layer thickness (cm)	Spacing (m)	Spacing (m)	Length (m)
Code for design of road tunnel (three lanes)	16–24	0.8–1.2	0.8–1.2	3.0–3.5
Guidelines for design of highway tunnel (four lanes)	25–28	0.75–1	0.75–1.0	3.5–6.0
Code for design of railway tunnel	20–23	Unquantified	1.0–1.2	3.0–3.5
Code for design of metro	No restrictions and recommendations on supporting parameters are given, "Shotcrete, steel arches or rock bolts should be used as the primary supporting means"			
Technical code for engineering of ground anchorages and shotcrete support	Unclear	/	0.5–1.25	Unclear
Norwegian method of tunneling	12–15	3–4	1.22–1.7	5

#### Investigation of other tunnel projects

**Figure 4:**
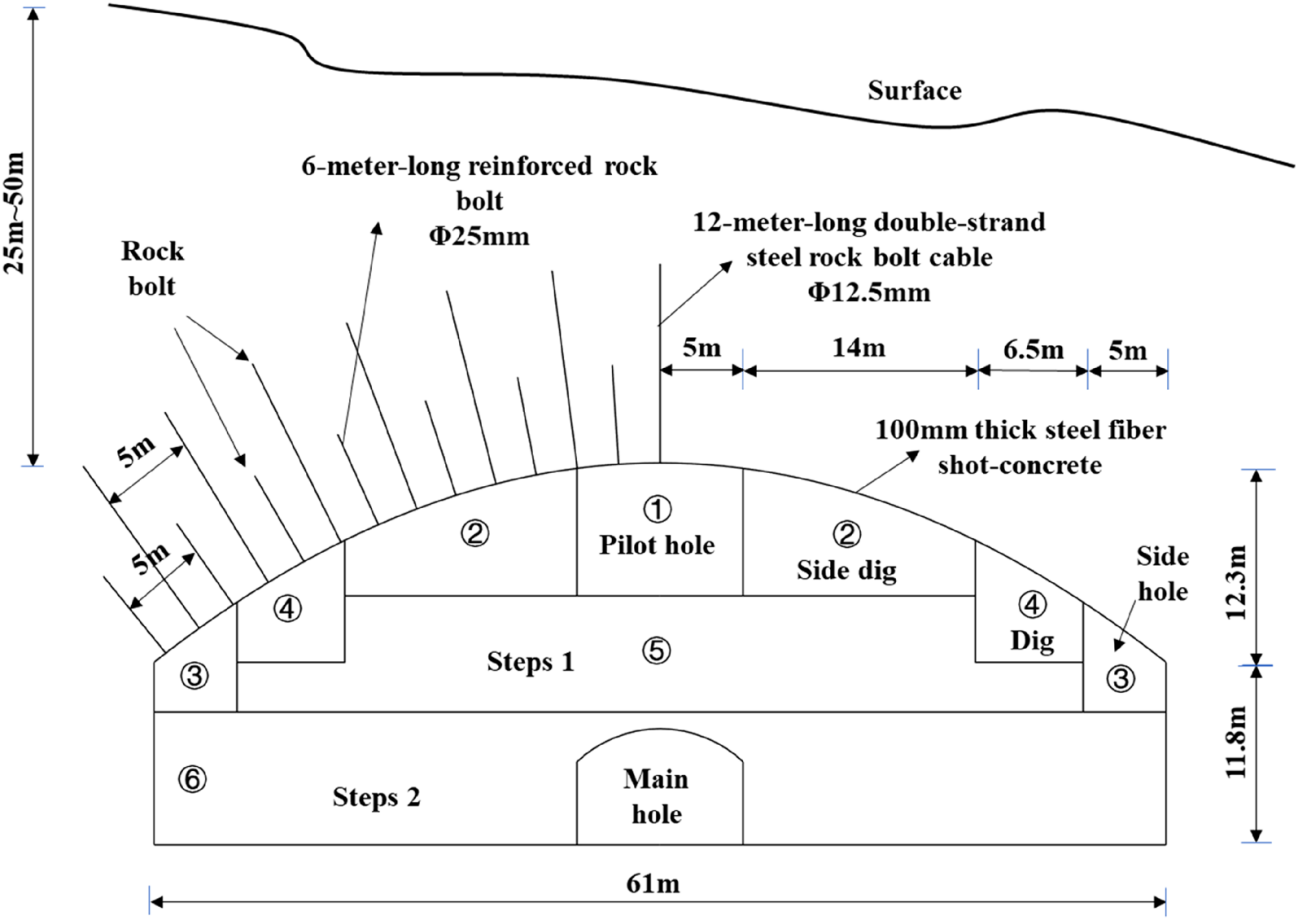
Layout of rock bolts on the roof of the Norwegian Olympic ice hockey stadium in gjøvik^18^.

The primary structure of Qingdao Metro test section (Qingfang Hospital Station) is a single arch long-span structure, the buried depth of the primary structure of the station is approximately 10 m, and the cavern is wide × height is 18.5 m × 14.0 m in the form of rock bolt and shotcrete support. Also, no supporting arch was set for surrounding rocks of grade III and IV. The bedrock of the Qingdao Jiaozhou Bay Subsea Tunnel consists of moderately and marginally weathered granite and volcanic rock. In the tunnel support scheme, the surrounding grade-III rock was not supported with a supporting arch. The Qingdao Taixi third road bifurcation tunnel has a span of 28 m and a burial depth of 14 m. No supporting arch was set in the surrounding grade-II and -III rock. A single layer lining is used for the Xizhen station of Qingdao Metro Line 1. The Norwegian Olympic ice hockey stadium in gjøvik has a span of 61 m, the overburden thickness is 25–50 m, and the rock bolt and anchor cable are 2.5 m × 2.5 m (circumferential × longitudinal) and are arranged alternately, as shown in Fig. 4. The systematic rock bolt was not constructed in the Xiang'an end field of the Xiamen subsea tunnel, an I-beam was not set in the surrounding rock of grade-III Baishan tunnel, and no supporting arch was set for the initial support of the Yabao tunnel. The surrounding rock of the granite stratum in Qingdao has good self-stability. Comparing these reports, we infer that the station should not set a supporting arch in the surrounding grade-III rock, and that the supporting arch spacing in the surrounding grade-IV rock should not be less than 1 m.

Based on these considerations, the traditional design method must be improved, and parameters must be optimized. Optimization is intended to improve the effectiveness of the rock bolt using prestress force to ensure construction quality by appropriately increasing the spacing between the arches and strengthening the support of key parts, such as the large arch foot area, sidewalls and junctions. The mechanism of the surrounding rock forming arch and the effect of the supporting structure are clarified; the work quantity of the arch rock bolt is reduced; the hollow grouting bolt is changed into a prestressed rock bolt; and the number and layout parameters of the rock bolts and supporting arches are optimized in this study.

## Principles of active support of high prestressed rock bolts

After the surrounding rock of the tunnel is excavated, radial stress loss easily damages the surrounding rock, which is not conducive to load bearing and stability. Using high prestressed rock bolts, the three-dimensional stress can be compensated for quickly and actively to enhance the integrity of the rock mass and form a combined load-bearing arch. Under the combined action of the free section, the rock bolting section, and the pallet, the prestress of the rock bolt diffuses into the tunnel’s surrounding rock, and a certain range of compressive stress areas is formed at the end of the rock bolt. The spreading range of the prestress enlarges, and the surrounding rock between the rock bolt and the rock bolt is effectively supported. As a form of active support, prestressed rock bolts can effectively control the continuous deformation of surrounding rock, make the support operate under the full stress–strain characteristics^19^, and maintain the integrity and self-supporting ability of surrounding rock.

**Figure 5:**
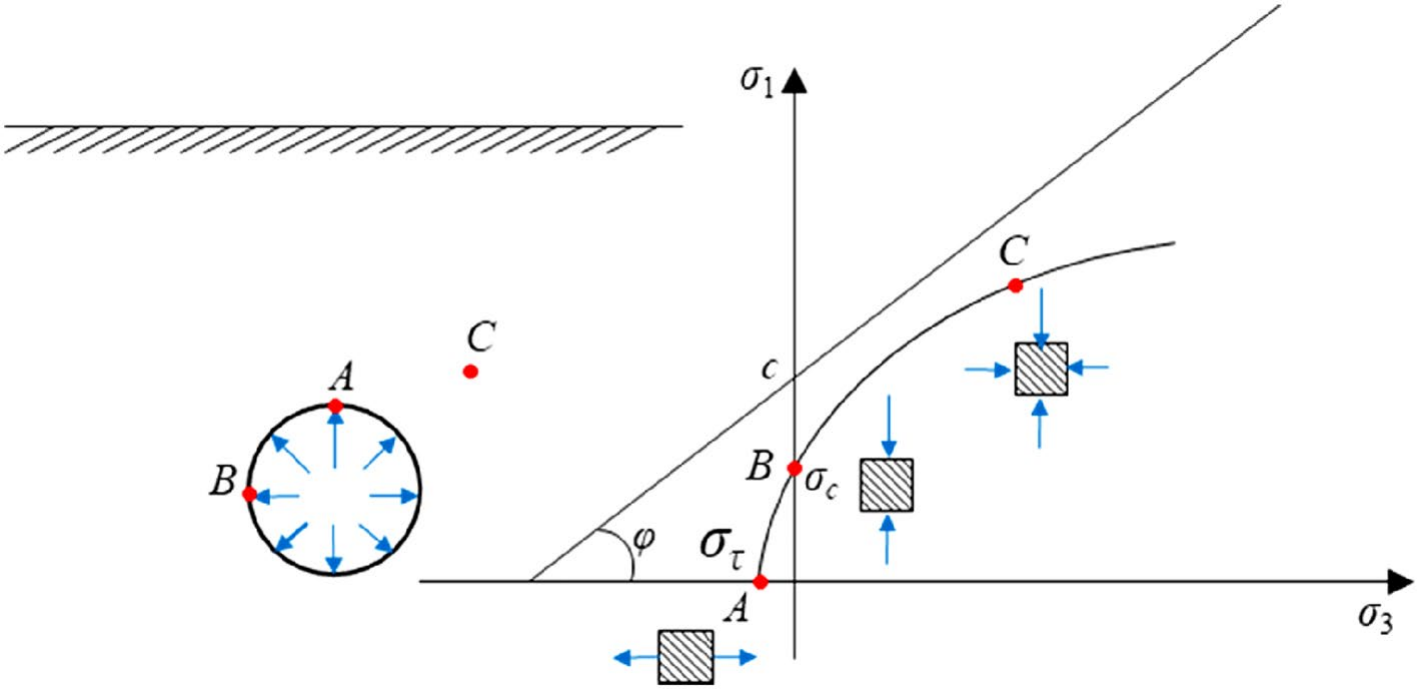
Stress state of surrounding rock after excavation.

**Figure 6:**
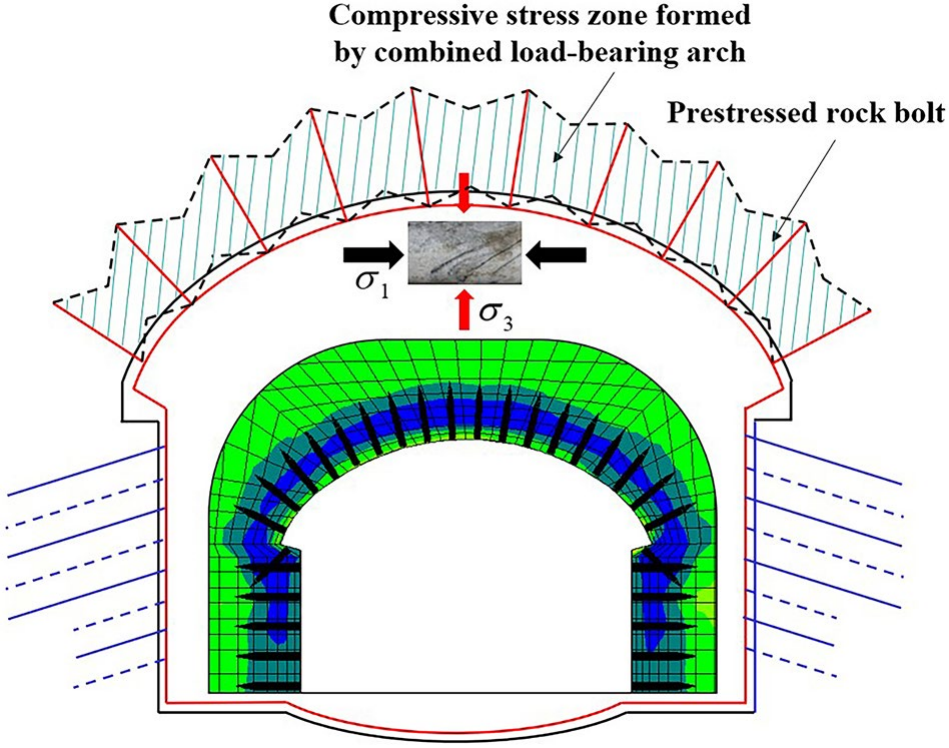
Combined load-bearing arch.

Under normal circumstances, there are three typical stress states after surrounding rock excavation (see Fig. 5). Due to tunnel excavation, the radial stress at point A disappears and is only subjected to unidirectional tension, which corresponds to point A of the Fig. 5. Due to the tunnel’s vacant surface, the stress of the surrounding rock in the radial direction on the right side of point B is 0, while the tangential direction of point B is subjected to unidirectional compression. Corresponding to point B of the Fig. 5, point C is far from the tunnel excavation surface and is nearly unaffected or not affected by the tunnel excavation and remains in the original rock stress state. The high-prestress support concept aims to restore the unloaded surrounding rock to the stable state of the surrounding rock or near the original rock stress state through the high-strength reverse restraint force to form a combined load-bearing arch^20–28^ (see Fig. 6). The combined support system of the shotcrete and supporting arch ensures the stability of the surrounding rock and achieves the purpose of tunnel support.

Compared with a traditional rock bolt (see Table 5), the high-prestressed rock bolt is an active support that has a higher bearing capacity, smaller borehole diameter, simple use of anchoring agent, higher strength, more convenient construction and a more controllable quality.

**Table 5:** Comparison table of traditional rock bolts and prestressed rock bolt.

Category	Specification/mm	Weight/kg/m	Yield strength/MPa	Tensile strength/MPa	Yield load/kN	Breaking load/kN
Hollow grouting bolt	Φ25	2.47	325	490	102	153
Prestressed bolts for mines	Φ20	2.46	500	670	157	210
High prestressed rock bolt	Φ18	2.00	625	950	159	242
High prestressed rock bolt	Φ16	1.58	625	950	126	191

## Optimization scheme and parameter design

### Overall technology roadmap of scheme optimization design

Based on engineering analogy, numerical calculations are used to increase the quantitative basis, and the numerical calculation results are corrected based on refined monitoring to realize dynamic design and ensure safety. We thus perform the first round of numerical simulations; determine the preliminary optimization scheme and implement it on site; and correct the formation mechanics parameters based on the field measurement results. The second round of detailed and in-depth numerical simulations are performed with the corrected values to study the surrounding rock arching mechanism and the influence law of supporting parameters, and form more reliable calculation results and supporting schemes.

### Calculation model

The three-dimensional fast Lagrange method is a numerical analysis method that is based on the three-dimensional explicit finite difference method and can simulate the three-dimensional mechanical properties of geotechnical materials or other materials. This method uses the explicit finite difference scheme to solve the control differential equation of the field and applies the mixed element discrete model, which can accurately simulate the material yield, plastic flow, softening and large deformation, particularly in the fields of material elastic–plastic analysis, large deformation analysis and simulation of the construction process.

**Figure 7:**
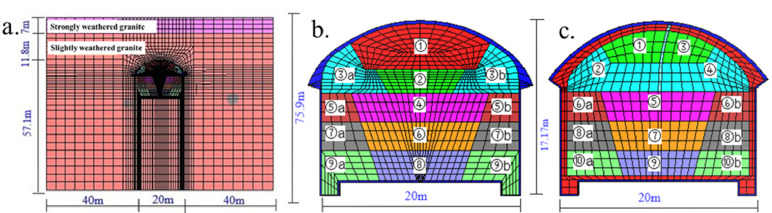
Three-dimensional model of the tunnel. (**a**) Front view of the surrounding rock and tunnel model, (**b**) PCM, and (**c**) SCM.

Based on typical sections, the stratum is simplified without considering the influence of tectonic stress. The Mohr–Coulomb yielding criteria is adopted by the modeling. The calculation model is 100 m in length, 75.9 m in height, and 1 m in thickness when the surrounding rock is grade IV1. The calculation model was established by CAD and imported into FLAC^3D^ and contained a total of 11,784 elements and 15,070 nodes. The stratum soil, spray layer and secondary lining are simulated by zone. The supporting arch uses beam elements, the middle wall uses beam + solid elements, the rock bolt uses cable elements, and the waterproof layer uses contact surface elements. The FLAC^3D^ model is shown in Fig. 7.

#### Surrounding rock parameters

According to the physical and mechanical property parameters of the rock and soil mass provided in the survey report, the surrounding rock parameters are used as the mechanical parameters required by the calculation model. Individual mechanical parameters not given in the survey report shall be determined according to the standard recommended values. Table 6 shows the parameter indices of the calculation model.

**Table 6:** Stratum parameters used in the numerical calculation (level-IV1 surrounding rock).

Stratum	Elastic modulus/GPa	Poisson ratio	Bulk modulus/GPa	Shear modulus/GPa	Cohesion/MPa	Internal friction angle/(°)	Unit weight/(kN/m^3^)
Strongly weathering granite	1.3	0.35	1.44	0.48	0.1	27	24
Marginally weathering granite	15	0.26	10.42	5.95	1	47	24

#### Supporting structure parameter selection

**Figure 8:**
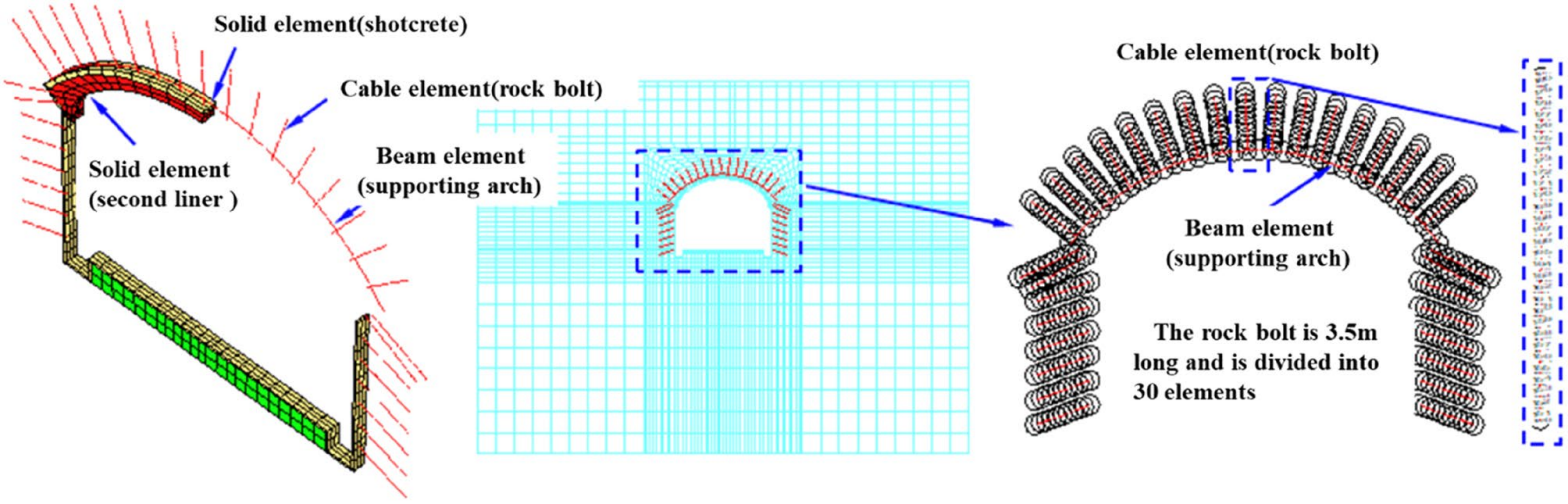
Three-dimensional model of supporting structure.

The shotcrete and secondary lining are simulated by solid elements, with an elastic modulus of 29.5 GPa, Poisson's ratio of 0.2, cohesion of 2.72 MPa and internal friction angle of 53.9°. The bulk modulus of the secondary lining is 18.6 GPa, the shear modulus is 13.9 GPa, the cohesion is 3.5 MPa, and the internal friction angle is 54°. The middle partition wall is simulated by a beam element and solid element. The shotcrete has a thickness of 300 mm, uses I22a section steel, has a longitudinal spacing of 0.5 m, has a cross-sectional area of 0.00421 m^2^, and has moment of inertia of 1.870 × 10^–8^. Selected parameters are shown in Tables 7 and 8, and the support structure model is shown in Fig. 8.

**Table 7:** Parameters of rock bolt and supporting arch.

Supporting member	Structural element	Cross-sectional area/m^2^	Length/m	Elastic modulus/GPa	Poisson ratio	Density/(kg/m^3^)	Tensile strength*/*MPa
Arch	Beam	19.6 × 10^–4^	–	200	0.25	7.85	–
Rock bolt	Cable	2.5 × 10^–4^	3.50	200	0.30	7.85	0.24

**Table 8:** Parameters used by the two construction methods.

Scheme	Grille		Arch rock bolt				Sidewall rock bolt			
Grade IV1	Initial support thickness/mm	Spacing/m	Length/m	Longitudinal distance/m	Ring distance/m	Prestress/kN	Length/m	Longitudinal distance/m	Ring distance/m	Prestress/kN
The original scheme of the SCM	350	0.5	3.5	0.5	1.5	0	3.5	0.5	1.5	0
The optimized scheme of the PCM	350	1	3.5	1	1.5	80	4	1	1.8	80

The contact surface element only transmits the normal stress, not the tangential stress. The SCM is provided with a waterproof board between the second lining of the arch and the initial support so that tangential action and normal tension are not transmitted between the two support structures. In the model, the contact surface element is used to simulate the waterproof layer, and the contact surface element is applied between the primary support and the secondary support to realize the characteristics that the waterproof layer only transmits normal stress and does not transmit tangential stress. The tangential stiffness, internal friction angle, tensile strength and cohesion of the contact surface are 0 and the normal stiffness is 2 × 10^10^ N/m.

### Result analysis

The results from the most dangerous conditions in the step-by-step construction (i.e., when the tunnel has been excavated and the second lining has not been installed) are considered below.

#### Vertical displacement

**Figure 9:**
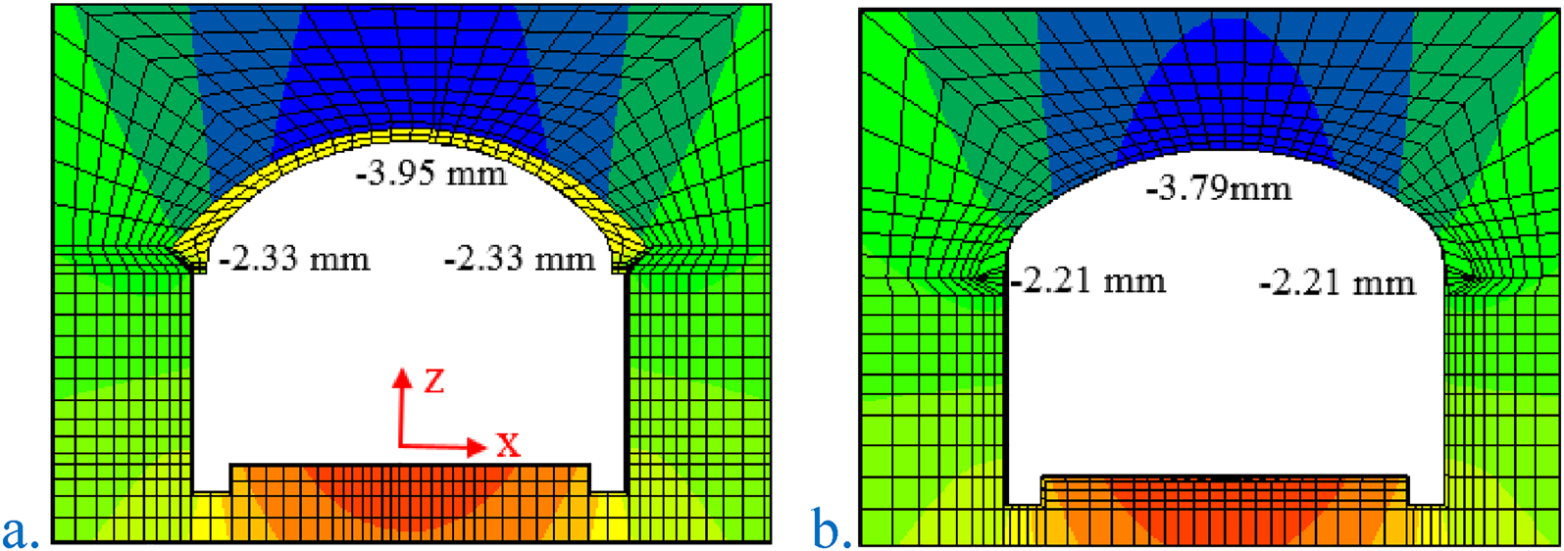
Vertical displacement cloud diagram of the two construction methods. (**a**) The original scheme of the SCM (IV1); (**b**) the optimized scheme of the PCM (IV1).

The vertical displacement cloud diagram of the two construction methods is shown in Fig. 9. When all excavation of the original scheme of the SCM was completed, the vertical displacement of the arch crown was − 3.95 mm, the vertical displacement of the left arch foot was − 2.33 mm, and the vertical displacement of the right arch foot was − 2.33 mm. When all excavation of the arch cover method was completed, the vertical displacement of the arch crown was − 3.79 mm, the vertical displacement of the left arch foot was − 2.21 mm, and the vertical displacement of the right arch foot was − 2.21 mm. The settlement of the arch crown caused by the arch excavation of the two construction methods accounted for 96.5% and 93.4% of the total settlement. With the same parameters, the vertical displacement of the arch crown of PCM was − 3.79 mm, and the vertical displacement of the arch crown of the two lining arch cover methods was − 3.95 mm. The arch settlement of PCM was 4.8% lower than that of the SCM.

#### Horizontal displacement

**Figure 10:**
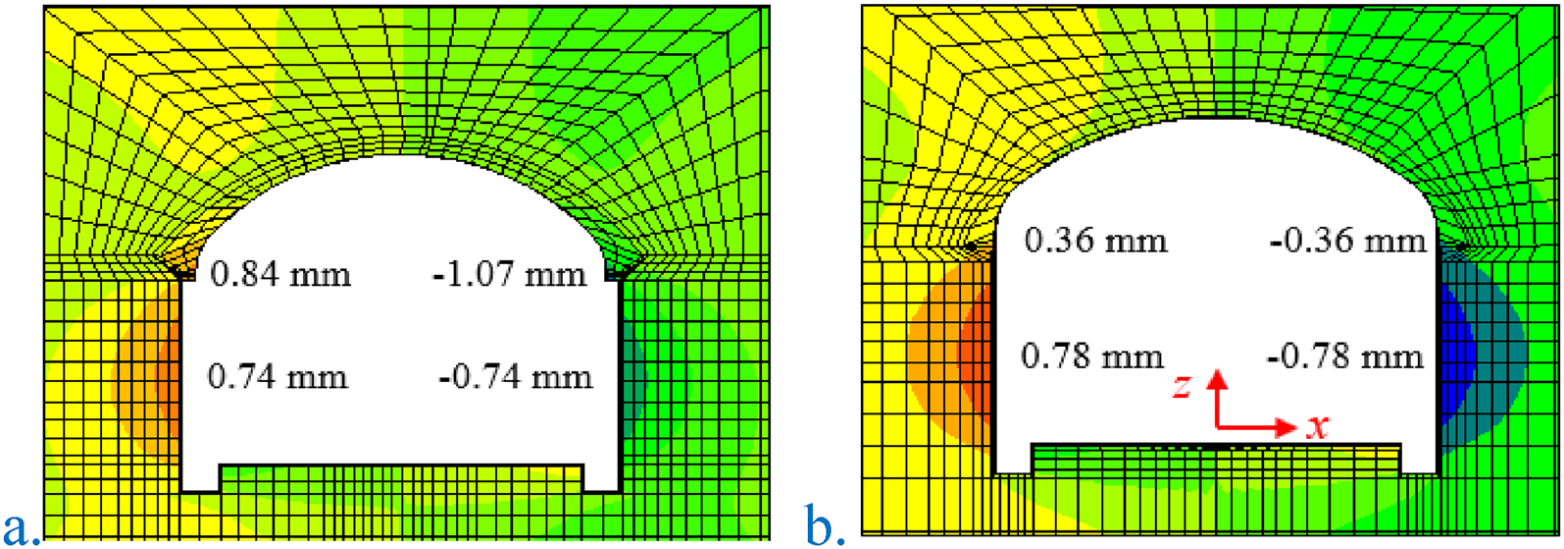
Horizontal displacement cloud diagram of the two construction methods. (**a**) The original scheme of the SCM (IV1); (**b**) the optimized scheme of the PCM (IV1).

The horizontal displacement cloud diagram of the two construction methods is shown in Fig. 10. When all excavation of the original scheme of the SCM was completed, the horizontal displacement of the left arch foot was 0.84 mm, the horizontal displacement of the right arch foot was 1.07 mm, and the displacement of the left and right sidewalls was 0.79 mm, both moving to the inner side of the arch. When all excavation of the arch cover method was completed, the horizontal displacement of the left and right arch feet was 0.36 mm, and the displacement of the left and right sidewalls was 0.78 mm, all moving to the inside of the arch.

#### Plastic zone and supporting structure force

**Figure 11:**
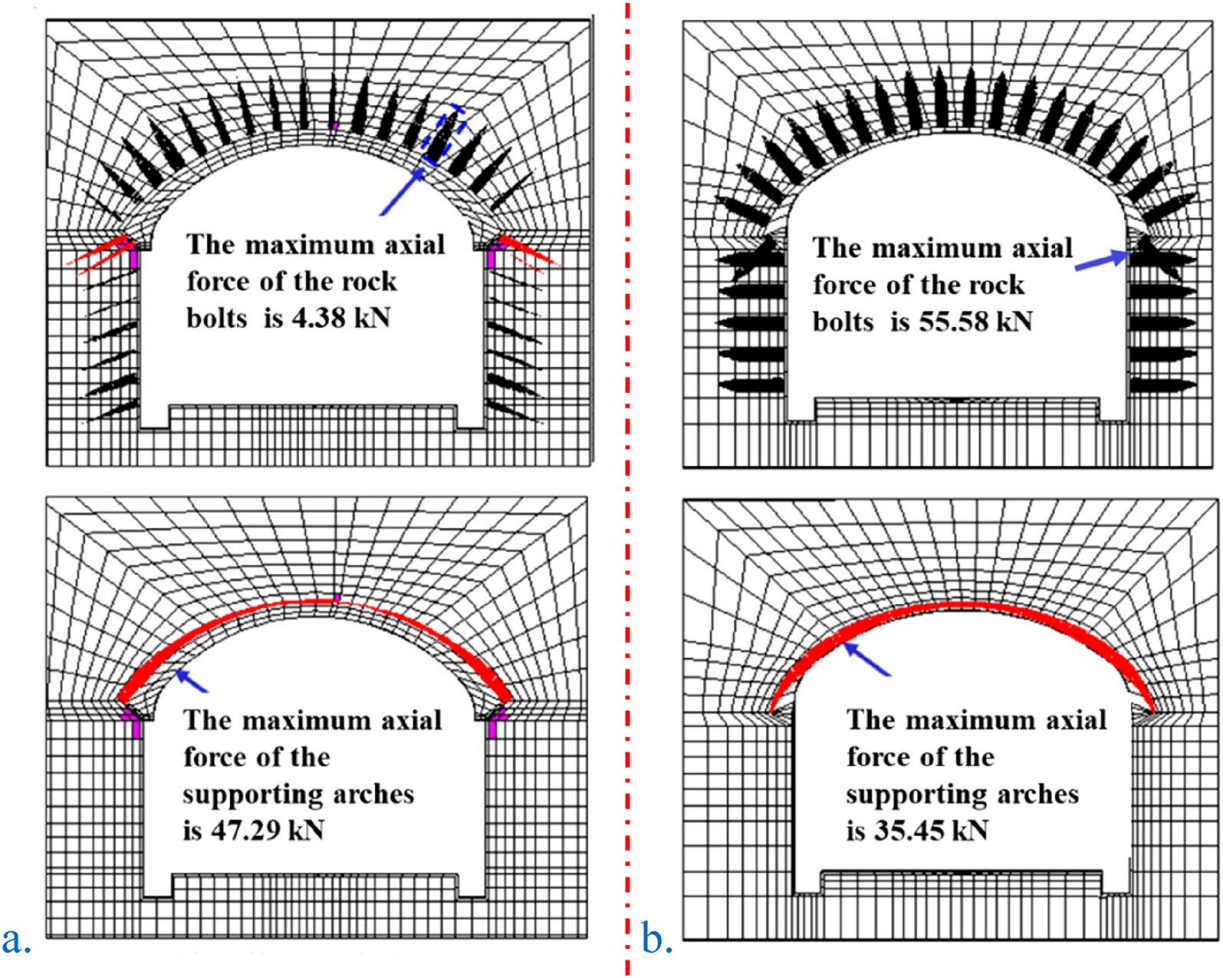
Two types of construction methods: plastic zone and supporting structure force diagram. (**a**) The original scheme of the SCM (IV1); (**b**) the optimized scheme of the PCM (IV1).

The force diagram of the plastic zone and supporting structure of the two construction methods is shown in Fig. 11 (black represents tension and red represents compression). The maximum axial force of the rock bolts in the original scheme of the SCM was 4.38 kN at the right shoulder, and the maximum axial force of the supporting arch was 47.29 kN. The maximum axial force of the rock bolt in the optimized scheme of the PCM was 55.58 kN, and the arch had a maximum axial force of 35.45 kN. The axial force of the rock bolt in the PCM increased markedly due to the active support. There is no plastic zone in the PCM, and the plastic zone area in the SC is 5.52 m^2^. The SCM included only self weight, and the waterproof layer was set between it and the surrounding rock. The weight acted on the arch foot; thus, there was a plastic zone. The surrounding rock of PCM had no plastic zone; the SCM had a slightly larger arch axial force due to the large settlement of the arch crown. Also, the distribution of the axial force of the arch in the two construction methods was different because the waterproof layer does not transmit the shear force. The bending moment of the arch was small and similar to that in the pure pressure state; and the axial force of the prestressed rock bolt markedly increased due to the active support.

#### Safety factor

**Figure 12:**
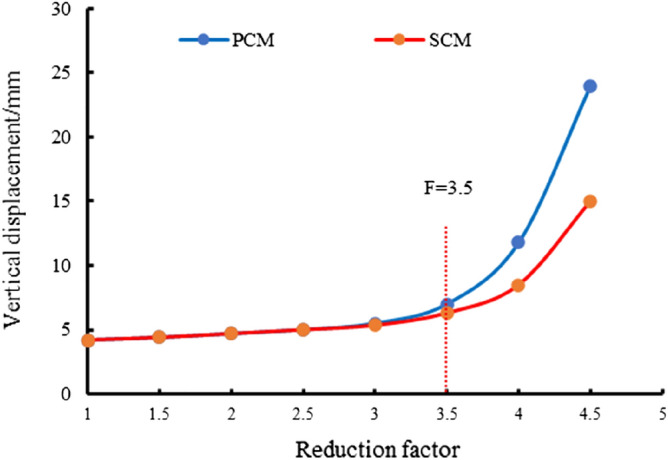
Curve of strength reduction method.

Figure 12 shows the results of the vertical displacement obtained by the two construction methods based on the strength reduction method with different reduction coefficients. Firstly, the inflection point of the correlation curve between settlement of tunnel arch crown and strength reduction factor is found. Then, the strength reduction factor corresponding to the inflection point is taken as the safety factor of the strength reduction method. The safety factor *F* of the settlement catastrophe point of the original scheme (SCM) and the optimization scheme (PCM) were approximately 3.5.

The displacement, supporting structure axial force, plastic zone area and safety factor statistics of the two construction methods are shown in Table 9. Considering the factors of force, construction and construction period, PCM should be used.

**Table 9:** Comparison of two construction methods.

Construction method	SCM	PCM
Arch crown settlement	3.95 mm	3.79 mm (marginally smaller)
Move the sidewall inward	0.74 mm	0.77 mm
Maximum axial force of rock bolt	3.14 kN	55.78 kN (significantly increased)
Maximum axial force of arch	51.27 kN	46.85 kN
Plastic zone area of surrounding rock	5.52 m^2^	0 (significantly reduced)

### Parameter design

The support structure scheme takes the calculation results of the unsupported tunnel of grade IV1 as a quantitative reference, performs a comprehensive design, and implements the differential design of the arch according to the monitoring data. For the arch spacing, while meeting the requirements of the specification, the convenience of construction should be considered, and the spacing should be appropriately increased with reference to engineering experience. Concurrently, the thickness of the spray layer should be appropriately increased to ensure rigidity and meet the high requirements of subway engineering risk prevention and control. For arch rock bolts, when the surrounding rock grade is good, the tensile stress area is primarily controlled. Level-III surrounding rock can reduce the rock bolt at the arch waist. Also, when the surrounding rock grade is poor, the arch tension stress area and the arch waist (shear) are controlled, and the plastic zone is dominated.

#### Analysis of unsupported tunnel

The tunnel is a shallow buried tunnel, the Poisson ratio is 0.26, and the ratio of vertical to horizontal stresses is approximately 2.85:1.

##### Analysis of large principal stress and small principal stress of unsupported grade-IV1 tunnel

**Figure 13:**
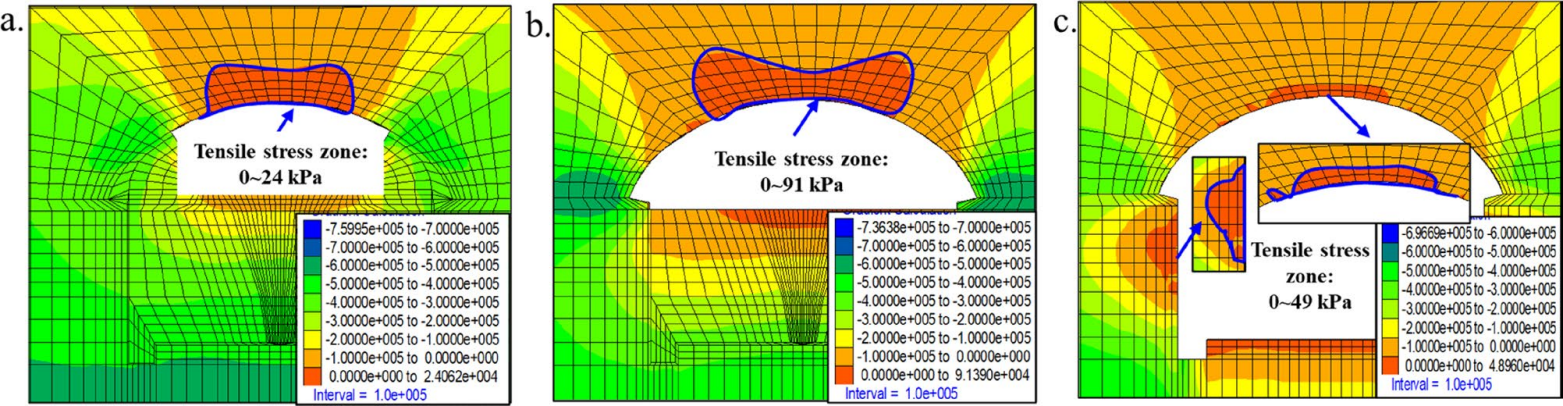
Small principal stress diagram. (**a**) Excavation of the upper steps in the middle of the arch, (**b**) excavation of the arch, and (**c**) all excavations.

When the excavation of the upper steps in the middle of the arch is completed, the tensile stress zone is located at the top of the arch crown with a maximum value of 24 kPa. When the arch excavation is completed, the tensile stress zone is located at the top of the arch with a maximum value of 91 kPa. Also, when all excavation is completed, the tensile stress area is located on the arch crown and sidewalls, and the small principal stress is shown in Fig. 13.

##### Analysis of tensile stress zone and plastic zone of an unsupported grade-IV1 tunnel

**Figure 14:**
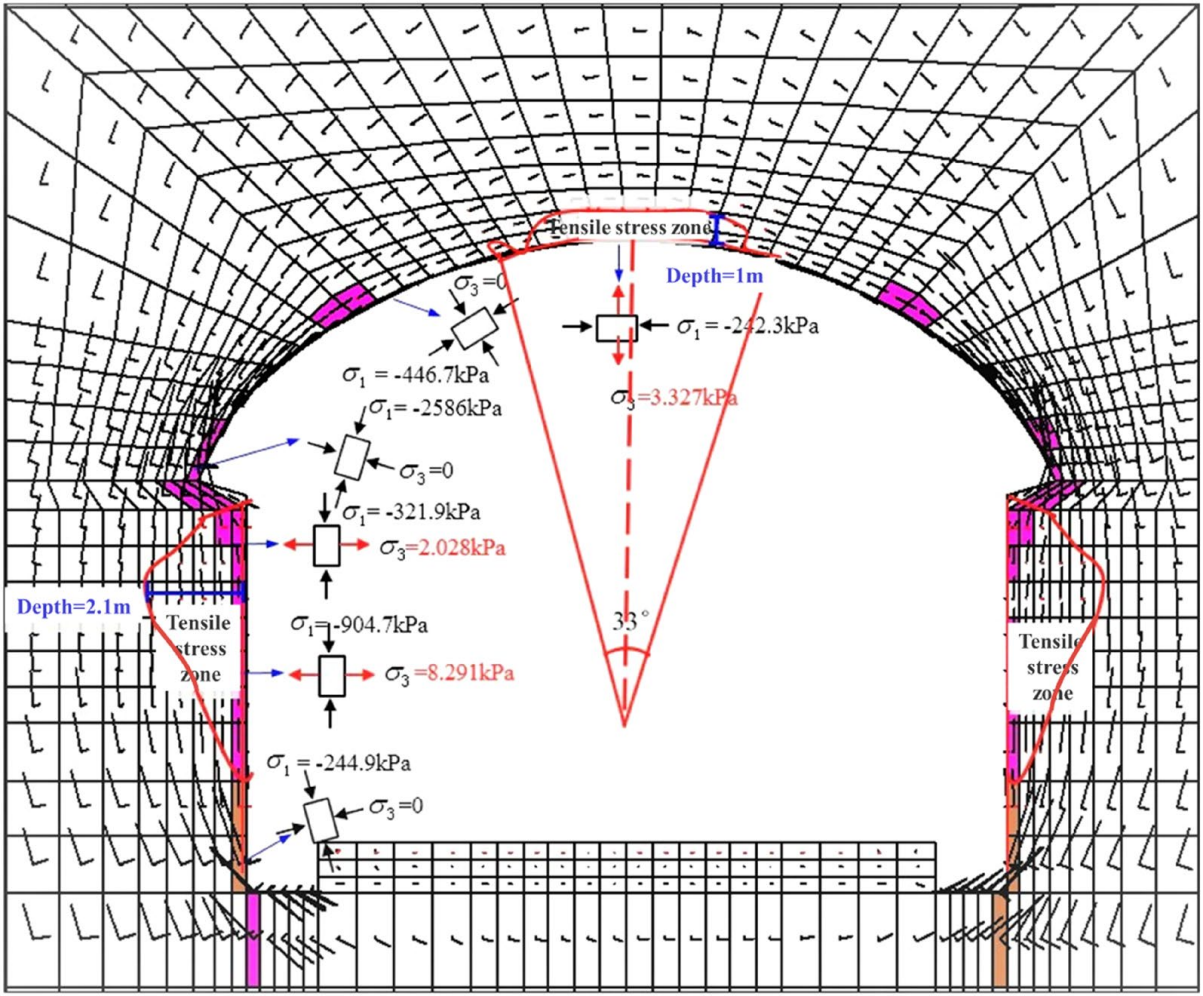
Principal stress vector diagram of an unsupported grade-IV1 tunnel.

Figure 14 is a vector diagram of the principal stress of the unsupported grade-IV1 tunnel. The direction of the line represents the direction of the principal stress, and the length represents the size. Black indicates compression, and red indicates tension. The tensile stress area is located on the arch crown and the sidewalls on both sides. The tensile stress depth at the arch crown is 1 m, the angle is 33°, and the small principal stress *σ*_3_ is 3.33 kPa; the tensile stress depth of the sidewalls on both sides is 3 m, and the maximum value of the small principal stress σ_3_ is 8.29 kPa; the plastic zone is located at the arch waist, the arch feet, sidewalls, and arch feet have significant plastic zones; the plastic zones are all shear plastic zones.

##### Calculation results of unsupported tunnels under different working conditions

**Figure 15:**
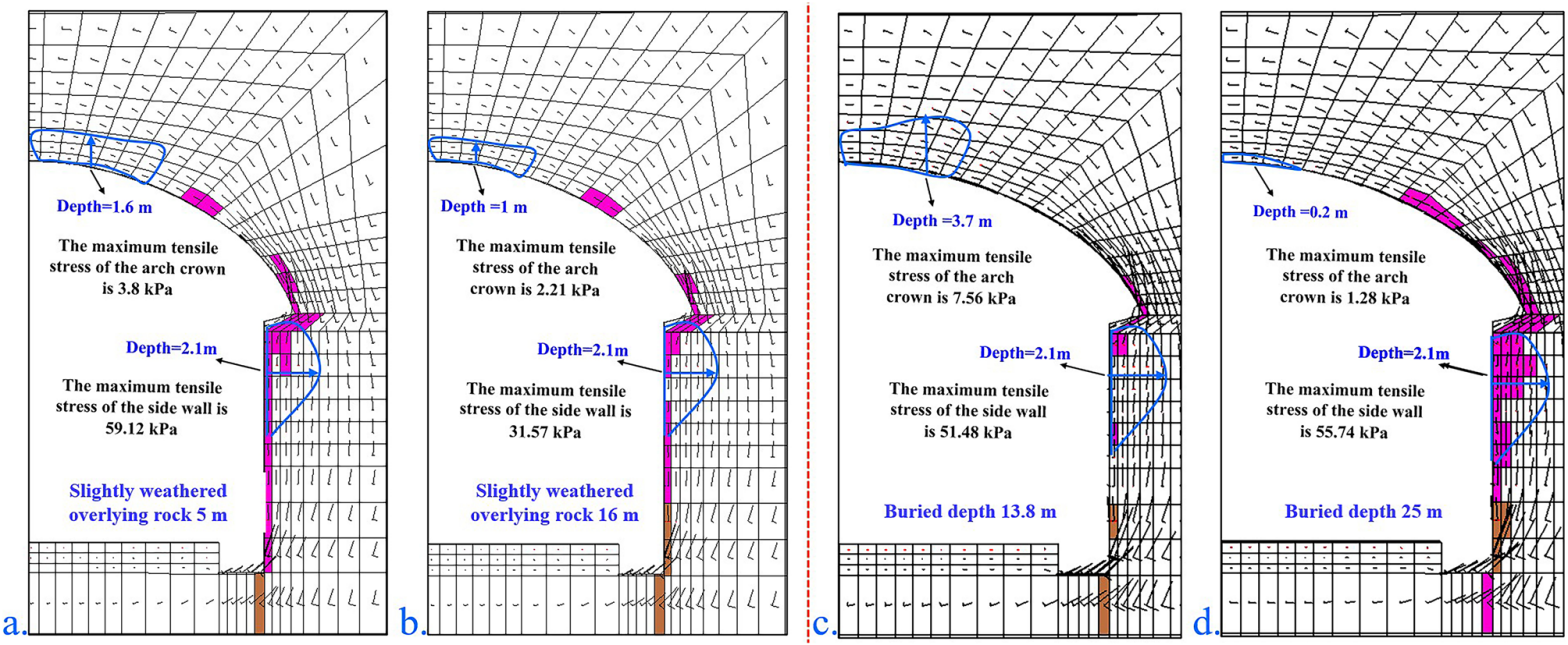
Principal stress vector diagram of the unsupported tunnel in level-IV1 surrounding rock under different working conditions. (**a**) slightly weathered overburden with a depth of 5 m; (**b**) slightly weathered overburden with a depth of 16 m; (**c**) buried depth of 13.8 m; (**d**) buried depth of 25 m.

Figure 15a and b shows the vector diagram of the principal stress of the cave with different thicknesses of the marginally weathered overburden. When the thickness of the marginally weathered overburden is 5 m, the plastic zone angle of the arch crown is 34°, the depth is 1.6 m, and the maximum tensile stress of the arch crown is 3.8 kPa. The depth of the plastic zone of the sidewall is 2.1 m, and the maximum tensile stress of the sidewall is 59.12 kPa. When the marginally weathered overburden is 11.8 m, the angle of the plastic zone of the arch crown is 33°, the depth is 1.5 m, the maximum tensile stress of the arch crown is 3.27 kPa, and the plastic zone of the sidewall has a depth of 2.1 m, and the maximum tensile stress of the sidewall is 57.21 kPa. When the breeze overburden is 16 m, the angle of the plastic zone of the arch crown is 30°, the depth is 1 m, the maximum tensile stress of the arch crown is 2.21 kPa, the depth of the plastic zone of the sidewall is 2.1 m, and the sidewall has a maximum tensile stress of 31.57 kPa. With increasing thickness in the marginally weathered overburden, the tensile stress zone of the arch crown is marginally reduced, the plastic zone of the arch foot is reduced, and the sidewall is basically unchanged.

Figure 15c and d shows the vector diagram of the principal stress of the burrows with different burial depths. When the buried depth of the grade-IV1 surrounding rock is 13.8 m, the plastic zone angle of the arch crown is 34°, the depth is 3.7 m, and the maximum tensile stress of the arch crown is 7.56 kPa. The plastic zone depth of the wall is 2.1 m, and the maximum tensile stress of the sidewall is 51.48 kPa. When the grade-IV1 surrounding rock is buried at 18.8 m, the arch plastic zone angle is 33°, the depth is 1.5 m, the maximum tensile stress of the arch crown is 3.27 kPa, and the plastic zone of the sidewall. With a depth of 2.1 m, the maximum tensile stress of the sidewall is 57.21 kPa. When the grade-IV1 surrounding rock is buried at a depth of 25 m, the plastic zone angle of the dome is 20°, the depth is 0.2 m, the maximum tensile stress of the arch crown is 1.28 kPa, and the plastic zone depth of the sidewall is 2.1 m. The maximum tensile stress of the sidewall is 55.74 kPa. When the grade-IV1 surrounding rock is buried at a depth of 34 m, the maximum tensile stress of the arch crown is 1.28 kPa, the depth of the plastic zone of the sidewall is 2.1 m, and the maximum tensile stress of the sidewall is 40.26 kPa. As the burial depth increases, the tensile stress area of the arch decreases, the tensile stress area of the sidewall remains unchanged, and the plastic area increases significantly, particularly the sidewall.

According to the results of step-by-step excavation and support, the statistical tables of the tensile stress area and plastic area of each surrounding rock level are shown in Table 10.

**Table 10:** Comparison of the tensile stress zone and plastic zone range of the surrounding rock of different grades.

Surrounding rock grade	Tensile stress zone range			Plastic zone range		
	Arch crown	Arched waist	Sidewall	Arch crown	Arched waist	Sidewall
Grade III	Range 36° depth 3 m	None	None	None	Depth 0.5 m	Near arch foot depth 1 m
Grade IV1	Range 33° depth 2.9 m	None	None	None	Depth 0.5 m	Locally 1 m deep
Grade IV2	Range 30° depth 2.8 m	None	None	None	Penetrate the arch foot to a depth of 1 m	Penetrate the arch foot to a depth of 1 m

##### Calculation results of unsupported tunnel under different prestresses

**Figure 16:**
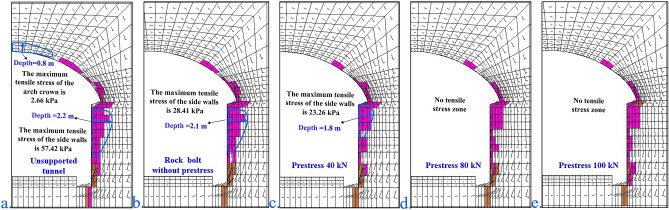
Rock bolts and prestressed plastic zone diagram of grade-IV2 pores. (**a**) unsupported tunnel; (**b**) rock bolt without prestress; (**c**) prestress of 40 kN; (**d**) prestress of 80 kN; (**e**) prestress of 100 kN.

Figure 16 shows a diagram of the rock bolt and prestressed plastic zone applied to the grade-IV2 unsupported tunnel hole. When there is no support, the plastic zone angle of the arch crown is 30°, the depth is 0.8 m, the maximum tensile stress of the arch crown is 2.66 kPa, and the depth of the sidewall is 3.2 m. The maximum tensile stress of the sidewall is also 57.42 kPa. When only a rock bolt (no prestress) is used, the depth of the sidewall is 2.1 m, and the maximum tensile stress of the sidewall is 28.41 kPa. When the prestress is 40kN, the maximum tensile stress of the sidewall is 23.26 kPa. When the prestress is 80kN, there is no tensile stress area. When the prestress is 100kN, there is no tensile stress area.

#### Parameter design

##### Supporting arches and shotcrete

Considering the calculation results as a quantitative reference, the comprehensive design is performed, and the spacing of the grid steel frame is first increased. Grid spacing is set to meet the specification requirements, considering construction convenience and engineering experience. Regarding the spray layer thickness, the grid spacing was expanded, and the spray layer thickness was appropriately increased to ensure rigidity and meet the high requirements of subway engineering risk prevention and control. The design spray layer thickness of the grade-III surrounding rock arch grid is 350 mm, and the grid spacing is 1.2 m. The design spray layer thickness of the surrounding rock of a grade-IV1 arch grid is 350 mm, and the grid spacing 1.0 m. The grade-IV2 surrounding rock arch’s grid thickness of the designed spray layer is 350 mm, and the grid spacing is 0.8 m.

##### Arch rock bolts

When the surrounding rock grade is of sufficient quality, the tensile stress area is controlled as the primary reason, and grade-III surrounding rock can reduce the arch waist rock bolt. When the surrounding rock grade is poor, we control the tensile stress area of the arch and the shear plastic area of the arch waist, and carry out differential design of the arch according to the monitoring data.

The calculated tensile stress zone and the plastic zone development depth are both less than 1 m, the measured loose circle is less than 1 m, and the rock bolt is not less than 3.5 m. It is feasible that the longitudinal spacing is the same as the supporting arches, and the circumferential spacing is 1.5 m and is not more than half the length. The longitudinal difference under different prestress levels is calculated. The results show that the greater the prestress, the greater the influence. There is basically no significant difference after 80KN, so the prestress design is 80 kN. The design length of the rock bolt for the arch of grade-III surrounding rock is 3.5 m, the quincunx layout is 1.5 m × 1.2 m (ring × longitudinal), and the prestress is 100 kN. The design length of the rock bolt for the arch of the grade-IV1 surrounding rock is 4.0 m, and the quincunx layout is 1.5 m × 1.0 m (ring × longitudinal), and the prestress is 100 kN. The design length of the rock bolt of the grade-IV2 surrounding rock arch is 4.5 m, the plum blossom-shaped arrangement is 1.5 m × 0.8 m (ring × longitudinal), and the prestress is 100 kN.

##### Arch feet and sidewalls

The level of the rock bolt angle is more favorable, and the construction can be properly inclined. The excavation of the arch foot reinforcement area strictly controls the blasting vibration. In terms of length, calculations show that a depth of 3 m is the boundary of the tensile stress zone of the unsupported tunnel. The length is considered to be 3.5 m or 5.5 m. The lengthened rock bolt area expands downward to the middle height of the sidewall. In terms of angle and force, the sidewall rock bolts should be as horizontal as possible. The arch foot rock bolts and sidewall rock bolts are counted from the arch foot, and 5 rows of 5.5-m rock bolts strengthen the support; the rest of the 3.5 m rock bolts are placed on a 1.5 m × 1.5 m grid. Special conditions, such as a broken zone and unfavorable structural surface of the sidewall due to excavation, should be strengthened locally.

**Figure 17:**
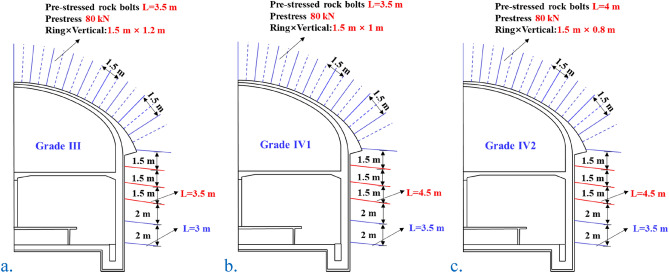
Supporting design drawings of surrounding rock of different grades.

The supporting design drawings of different surrounding rock levels are shown in Fig. 17.

#### Verification of optimization scheme effect

**Figure 18:**
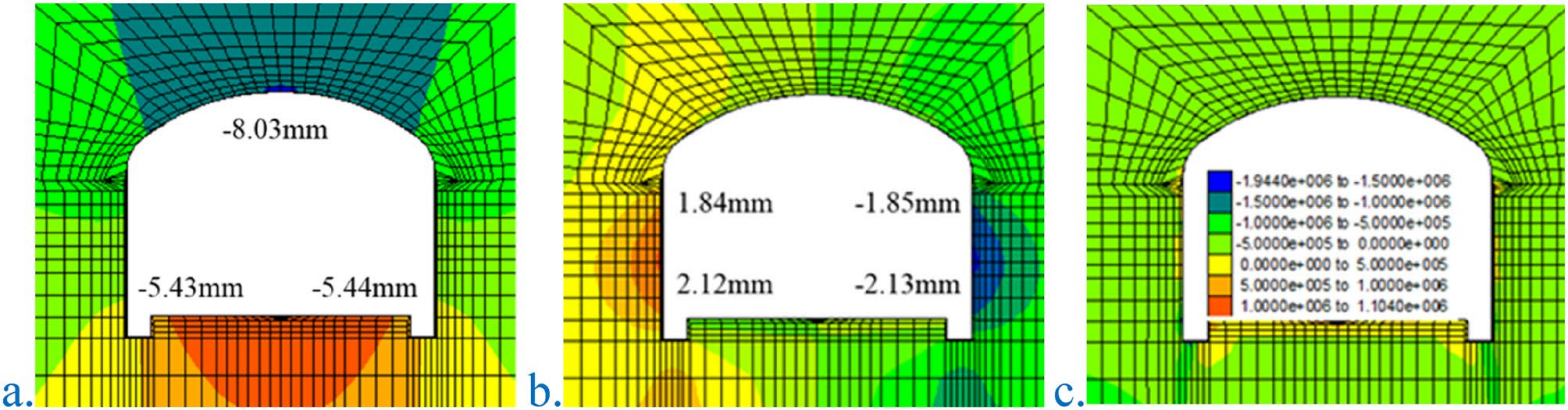
Simulation results of the level-IV2 surrounding rock. (**a**) Vertical displacement; (**b**) horizontal displacement; (**c**) small principal stress.

The effect of the optimization scheme is verified. Figure 18 shows the simulation results with grade-IV2 surrounding rock. There are a few tensile stress areas on the surface of the arch and the sidewall, and the stress is relatively small due to the active support. After adopting the optimized scheme, the surrounding rock tensile stress zone disappears, the plastic zone is controlled, and the prestressed bolt has a significant active support effect.

## Field implementation and monitoring feedback

### Field implementation

#### Rock bolts installation process

**Figure 19:**
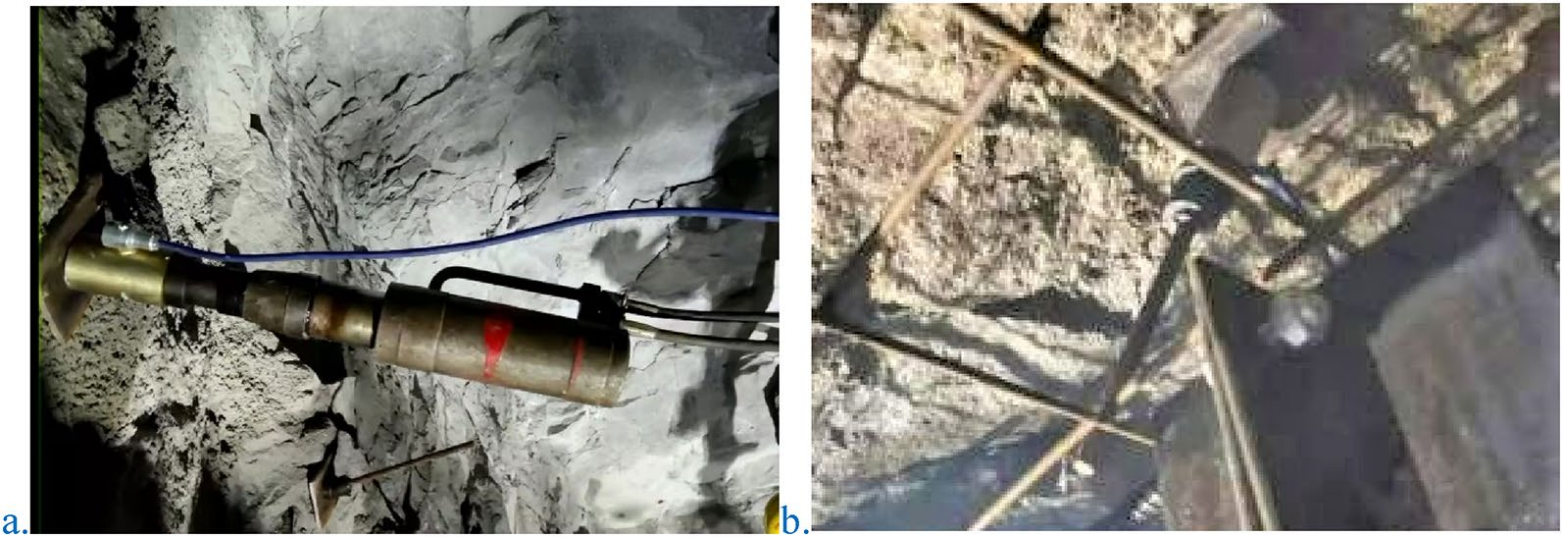
Real scene of the tensioning machine and the rock bolts. (**a**) Tensioning machine; (**b**) rock bolts.

After tunnel excavation, the position of high prestressed rock bolt shall be determined after necessary safety measures are taken. After drilling and cleaning the hole, install the resin anchoring agent first, then install the rock bolt, and then mix the resin anchoring agent to bond the rock bolt with the surrounding rock^29–31^. Next, install the tray and nut^32,33^, and finally tension the rock bolt through the tensioner to complete the installation of high prestressed rock bolt, as shown in Fig. 19. The new type of bolt support adopts a mineral resin rock bolting agent to bolt high-prestressed bolts. The rock bolting length is 1.2 m, and the average setting time of the resin rock bolting agent is approximately 20 s. The solidification time realizes the goal of quickly mobilizing the self-carrying capacity of the surrounding rock and avoids the expansion of the collapsed arch^34^. Compared with hollow grouting bolts^35–37^, high prestressed bolts can be applied with a prestress of 150–200 kN. After resin rock bolting, a tensile machine is used to quickly apply high prestress. The high prestressed bolts can interact with the surrounding rock in time as soon as possible to bear the pressure of the surrounding rock. The high prestressed rock bolt and the W steel belt (or reinforced ring) are arranged in the circumferential direction of the tunnel, supplemented by the longitudinal connection of the flexible net, and the high prestressed rock bolt rib arch formed after spraying concrete can markedly improve the integrity of the spray layer and hoop tensile strength to achieve full coverage of the tunnel excavation surface support. This process can also increase the rate of construction and reduce the support cost. Using high prestress and active support to the high prestressed bolts, the longitudinal spacing of the rock bolt rings is optimized accordingly, and the preliminary calculation reduces the number of bolts by 20%.

#### Rock bolts tension and locking process

When the strength of the rock bolt consolidation body reaches 75% of the design strength and is not less than 15 MPa, the rock bolt can be tensioned and locked. The tension-type steel strand rock bolt should use integral tension and locking of the steel strand harness. Before the rock bolt is locked, the rock bolt is pretensioned and loaded gently. The tensile force of the rock bolt during locking should consider the loss of prestress during locking, and the amount of prestress loss should be determined by testing the tensile force of the rock bolt before and after locking. Without test data, the tensile force of the rock bolt during locking may be 1.1 of the locking value. Rock bolt locking should consider the prestress loss caused by the tension and locking of adjacent rock bolts. When the rock bolt prestress loss is high, it should be locked again.

### Field monitoring

During the construction of the subway, the deformation and internal forces of important underground and ground structures (structures) were monitored, including ground roads around the subway structure project and construction along the line, to provide timely and reliable information for all parties involved in the construction for evaluation safety and implementation of subway engineering during construction.

The impact of construction on the surrounding environment and timely and accurate forecasts of potential hazards or accidents may endanger construction safety and the surrounding environment so that effective measures can be performed quickly to eliminate hidden hazards and avoid accidents.

#### Monitoring items

The specific monitoring items of the primary structure include arch crown settlement, clearance convergence, the force of the supporting arches, the axial force of rock bolts, the force of shotcrete, etc. Their measurement points are located in the same section, which is convenient for each comparison and verification of item data. The monitoring items are shown in Table 11.

**Table 11:** Summary of monitoring items.

No.	Monitoring item	Monitoring object	Instrument	Instrument details
1	Arch crown settlement	Tunnel structure and surrounding environment	Total station	BOR-EX drilling type multipoint displacement meter
2	Clearance convergence	Initial support structure of tunnel	Total station	/
3	The force of the supporting arches	Axial force of the supporting arches at the arch part	Supporting arch axial force instrument	Model: MAS-STG-25 Range: pull 200 MPa, pressure 200 MPa Accuracy: 0.07% Dimensions: Φ40 × 200
4	The force of shotcrete	Strain of shotcrete	Shotcrete strain instrument	Model: MAS-EM-30 Range: Pull 3000 με, pressure 3000 με Accuracy: ≤ 0.2% Dimensions: Φ120 × 26 × 38
5	Axial force of rock bolt	Axial force of rock bolt	Rock bolt axial force instrument	Range: (0–400) kN Accuracy: 0.1 kN Dimensions: Φ80 × 54
6	Ground settlement	Existing buildings within the affected area	Precision level	/

#### Monitoring section design

**Figure 20:**
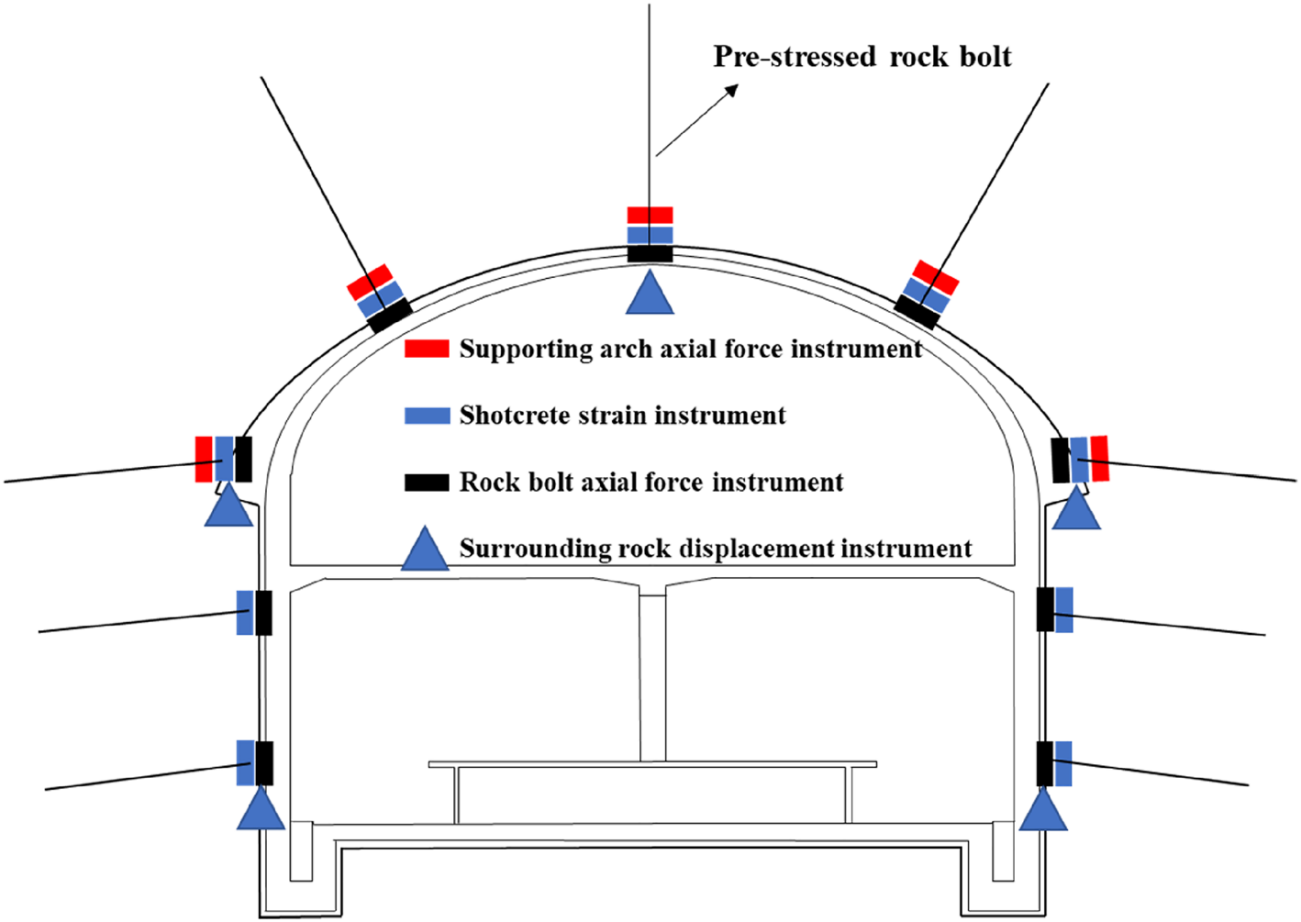
Type and layout of monitoring equipment.

The supporting arch axial force instrument monitors the arch axial force, the shotcrete strain instrument monitors the concrete strain, the rock bolt axial force instrument monitors the rock bolt axial force, and the surrounding rock displacement instrument monitors the displacement in the cave. The type and layout of monitoring equipment are shown in Fig. 20.

#### Monitoring equipment and use

##### Equipment

The instruments and equipment used on site are commonly used and reliable, and the quantity meets the requirements of monitoring work. A verification certificate was submitted for the monitoring equipment, and the verification (calibration) element of the instrument and equipment had national verification and qualification. The verification certificate was stamped with the official seal of the verification element, and the instrument was used within the validity period. After the installation of field monitoring instruments and equipment (see Table 12), we tested and calibrated the instruments, and recorded the initial values of the various instruments and equipment of the observation system under working conditions. All monitoring instruments and equipment were regularly inspected and maintained to ensure good working conditions.

**Table 12:** Primary monitoring instruments.

No.	Monitoring items	Requirements for the layout of measuring points	Instruments and equipment	Monitoring objects
1	Clearance convergence	Set on the same cross-section as the settlement of the arch crown, and horizontal survey lines are arranged for each pilot tunnel	Total station	Tunnel sidewall displacement
2	The force of the supporting arches	Points should be arranged in key parts such as arch crown, arch waist, arch foot, wall, wall toe, inverted arch, etc.; each representative section shall have no less than 7 measuring points	Reinforced axial force instrument	Axial force of arch grille steel frame
3	The force of shotcrete	Concrete components can be monitored with concrete strain gauges, etc., and one section should not be less than 11 measuring points in each representative section	Shotcrete strain instrument	Strain of shotcrete
4	Axial force of rock bolt	Set 1 section for each representative lot, and each section shall have no less than 7 measuring points	Axial force instrument	Axial force of rock bolt

##### Instrument installation

1. Installation of reinforced axial force instrument (supporting arches): When making the grid arch, the rebar meter is typically directly welded to the steel bar to be tested in pairs. The rebar meter has sufficient length to prevent damage to the internal strain. However, care was taken to ensure that the center part of the rebar gauge is not too hot because the coil bobbin and protective epoxy resin may melt. To prevent this from happening, it is necessary to place wet rags near the weld and the middle part of the rebar gauge during welding. The rebar meter installation and automatic monitoring equipment are shown in Fig. 21.
2. Installation of the shotcrete strain instrument: Concrete strain gauges were installed between the two grids using tie straps to fix the strain gauges onto the steel mesh. The sensor could not be welded because it would have been damaged. Therefore, the installation of the sensor was performed after all welding was completed. To avoid consolidation of the primary shotcrete and the surface strain gauge of the steel mesh, which will affect the test results, a protective cover device was used to protect the strain gauge.
3. Measuring method.

**Figure 21:**
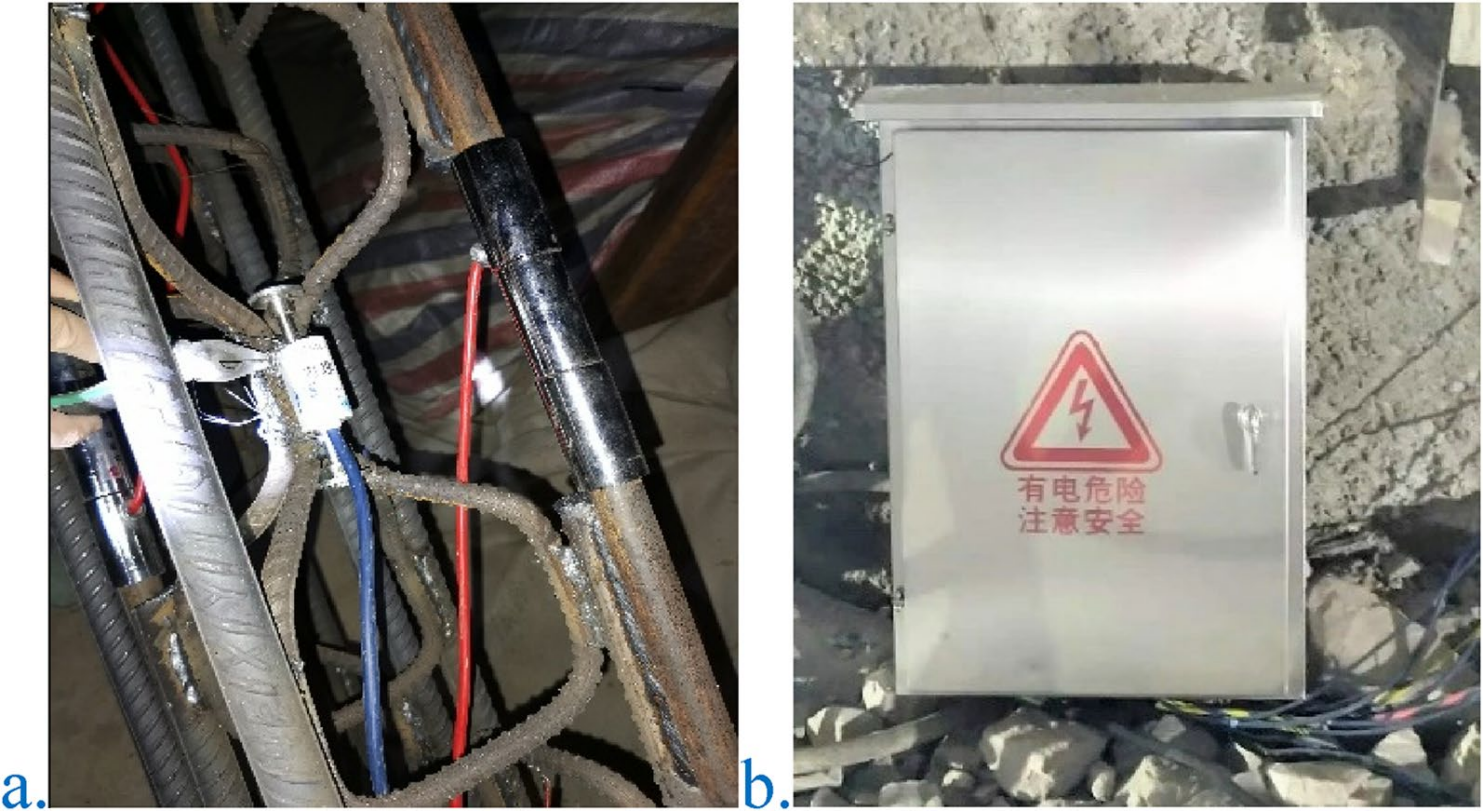
Actual view of the installation of the reinforcement meter and automatic monitoring instrument.
  A vibrating wire multifunction reader was used for measurement, a frequency meter was used to measure the frequency of the rebar meter, and the change in axial force was calculated according to the formula $P=K*({F}_{0}^{2}-{F}_{i}^{2})$, where *K* is the calibration coefficient, *F*_i_ is the test frequency, and *F*_0_ is the initial frequency. The calculated axial force *P* is the axial force of the supporting arches, and the stress of a single steel bar can be obtained by calculating the axial force. The formula is $\sigma = P/A$, where *A* is the cross-sectional area of a single steel bar of the supporting arches.
  Similarly, the vibrating wire multifunction reading instrument is used to measure the strain of the concrete strained by the frequency meter, and the change in strain is calculated by the formula $P=K*({F}_{0}^{2}-{F}_{i}^{2})$, where *K* is the calibration coefficient and *F*_i_ is the test frequency, *F*_0_ is the initial frequency, and the stress of the concrete can be obtained by calculating and measuring the concrete strain.
4. Monitoring frequency.
  The monitoring frequency of this project is based on the principle that it can systematically describe the important changes of the monitored item without omitting its change time.
  The monitoring frequency accounts for the engineering level, different construction stages, and changes in the surrounding environment and natural conditions. The monitoring frequency is as follows: when $L\le 2B$, the monitoring frequency is once a day; when $2B<L\le 5B$, the monitoring frequency is once every two days; when $L>5B$, the monitoring frequency is once every three days. Where, *B* represents the tunnel excavation width and *L* represents the horizontal distance from the excavation surface to the monitoring point. When the monitoring value is relatively stable, the monitoring frequency can be appropriately reduced, and when abnormal conditions or poor geological conditions occur, the monitoring measurement frequency can be increased.

#### Monitoring data

**Figure 22:**
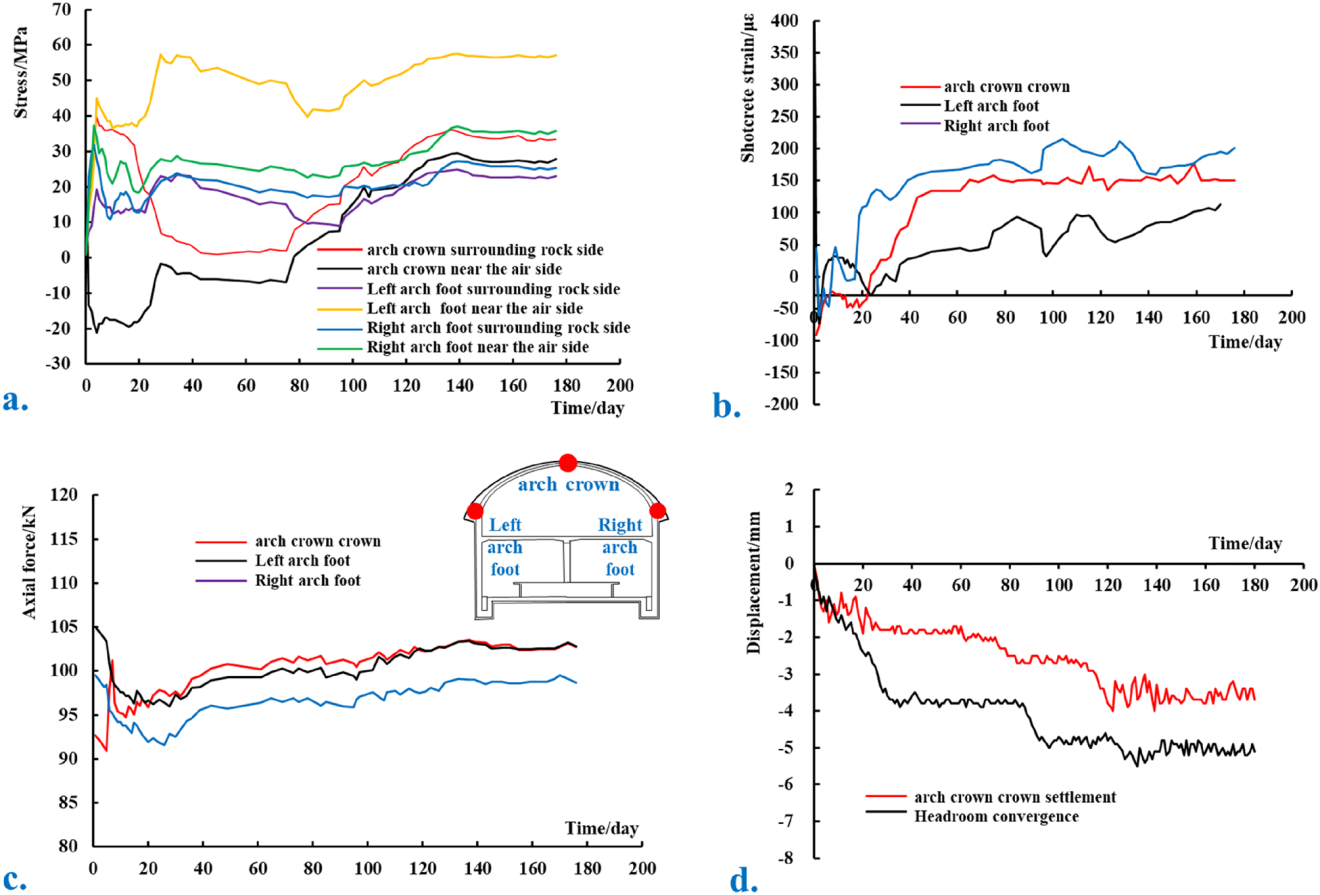
Field monitoring data diagram of a certain section of the primary section. (**a**) Stress of supporting arches; (**b**) strain of shotcrete; (**c**) axial force of rock bolts; (**d**) displacement of surrounding rock.

During field monitoring, the reinforced axial force instrument is used to measure the stress of the steel bar of the supporting arches, where a positive value is compressive, and a negative value is tensile. The shotcrete strain instrument is used to measure the shotcrete strain after the initial spraying, where a positive strain is compressive, and a negative strain is tensile. The axial force of the prestressed rock bolt is measured by the rock bolt axial force instrument, where a positive axial force is tensile, and a negative force indicates pressure. A total station is then used to measure arch crown settlement, where a negative value indicates settlement, and headroom convergence, where a negative value indicates convergence toward the empty surface. Through a period of field monitoring, a large amount of data has been obtained, and the data of a certain section of the primary section are shown in Fig. 22.

Comprehensive analysis is performed by combining field monitoring results and numerical simulation results. The stress of the supporting arch reinforcement is primarily compressive, and the maximum compressive stress appears in the left arch foot area, which is approximately 57.5 MPa, far lower than the yield strength of 400 MPa, and tends to be stable in approximately 30 days. The axial force of prestressed rock bolts is primarily under tension, and the maximum value is approximately 103 kN. After monitoring the initial prestress loss, there is a small decrease that stabilizes after approximately 10 days. The axial force of the rock bolts is greater than the numerical calculation result, and the active support effect is significant. The strain of the shotcrete is primarily compressive strain, and the maximum strain occurs in the left arch foot area, which is approximately 215 με. The strain change is small, and the maximum strain is less than the ultimate compressive strain. The axial force of the supporting arch reinforcement, the axial force of the rock bolts, and the strain of the shotcrete are all less than its yield limit.

With the increase of time, the settlement of arch crown increases rapidly at first, then slowly, and finally tends to be stable. The maximum settlement of the arch crown is approximately 4 mm, which is similar to the numerical simulation results. This shows that the new technology is more consistent with the actual situation on site and reduces the occurrence of engineering accidents.

**Figure 23:**
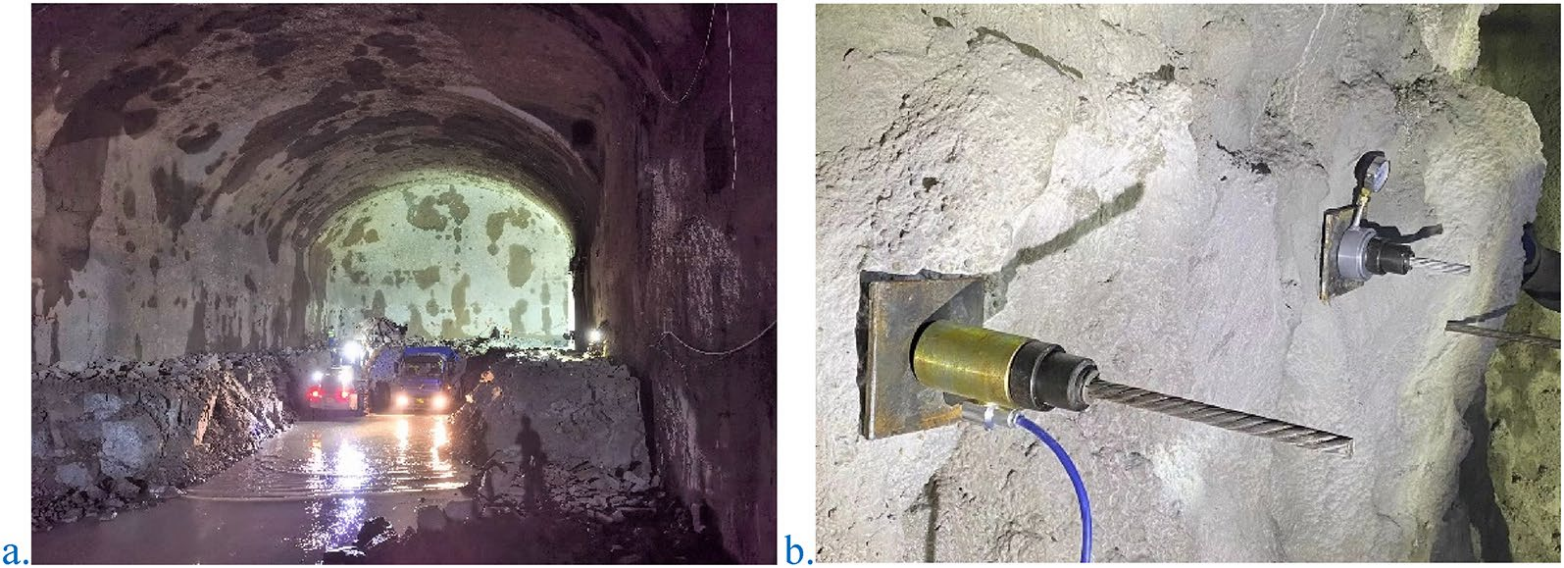
Real view of the tunnel.

Based on observations in the field (see Fig. 23), no major deformation occurred in the arch crown and other positions. The monitored value is within a safe range, and there is a certain safety reserve.

Compared with the existing subway line (see Table 13), the underground excavation subway station of Line 6 is compared with the abovementioned Hengshan Road Station, Zhiquan Road Station, Nanchang Road North Station, Jiadingshan Station, etc., which are similar in grade to the surrounding rock and the excavation span. The settlement of the arch crown is reduced by approximately 3–8 mm (a reduction of approximately 44–54%), which indicates that the high prestressed bolt system can effectively control the continuous deformation of the surrounding rock and is conducive to maintaining the integrity of the surrounding rock and giving full play to the self-supporting capacity of the surrounding rock. This method is more conducive to blasting organization and smooth blasting effects, and the construction period is shortened by 20–40 days.

**Table 13:** Summary of existing line data.

Site name	Hengshan road station of line 1	Zhiquan road station of line M2	Nanchang road north station of line 8	Jiadingshan station of line 8	Line 6 (six stations)
Construction method	SCM	SCM	SCM	SCM	Active support
Excavation method	Double sidewall pilot pit method	Double sidewall pilot pit method	Double sidewall pilot pit method + CD method	Double sidewall pilot pit method	Annular step method
Buried depth	The buried depth of the arch crown is approximately 18.7–16.5 m	The buried depth of the arch crown is approximately 13–18 m	The buried depth of the arch crown is about 14.26–15.73 m	The buried depth of the arch crown is approximately 12.3–21.8 m	The buried depth of the arch crown is approximately 16.3–33.2 m
Excavation width of arch cover	22.1 m	23.06	22.3 m	23.12 m	21.4 m
Surrounding rock conditions	III–V	III–V	III–IV	III–IV	III–IV2
Settlement of the arch crown after the excavation of the upper step is completed	8–12 mm	3.3–9.9 mm	5–15 mm	5–15 mm	3–6 mm

## Conclusion

1. Engineering conditions are analyzed, the traditional scheme construction method is standardized, and engineering analogy and paper investigation are considered. Results show that the original support scheme is conservative and must be optimized, and the optimization produces a highly prestressed rock bolt support system. Active support is used, and an optimized scheme design is performed. The effect of rock bolts is improved using prestress to ensure the construction quality of rock bolts, appropriately increasing the spacing between supporting arches, and strengthening the support of key parts such as the large arch foot area, sidewalls and junctions.
2. The support mechanism of high prestressed rock bolts was clarified, a high prestressed support system was established, and numerical calculation and analysis of the support system were performed. The results show that after the optimization scheme is used, the maximum axial force of rock bolt increases significantly from 3.14 to 55.78 kN; the plastic zone decreases significantly from 5.52 m^2^ to 0. These show that the optimization scheme can improve the stress state of surrounding rock more obviously, the rock bolt effect is significantly improved, and the active support effect of high prestressed rock bolt is high.
3. Field monitoring results show that the settlements of the arch crown are concentrated in 2–5 mm and the deformation rates are less than 0.5 mm/day. The supporting arches, shotcrete, and rock bolts are all less than the yield strength and have some safety reserve. Field monitoring indicates that the new support system improves the stability of the surrounding rock, has a lower cost, and is faster to construct. These results verify the safety and rationality of the new support system, which can be used as a reference for similar projects.
